# Photoelectrochemical comproportionation of pre-treated PET plastics and CO_2_ to formate†

**DOI:** 10.1039/d5ee00689a

**Published:** 2025-04-11

**Authors:** Yongpeng Liu, Celine Wing See Yeung, Erwin Reisner

**Affiliations:** a Yusuf Hamied Department of Chemistry, University of Cambridge Cambridge CB2 1EW UK reisner@ch.cam.ac.uk

## Abstract

Pairing plastic waste reforming and carbon dioxide (CO_2_) utilisation to produce chemical energy carriers provides an attractive means to mitigate waste and create value, but challenges persist in achieving selective product formation, separation and overall device integration. Herein, we present an organic–inorganic photoelectrochemical (PEC) tandem device that enables the solar-powered comproportionation of plastic waste and CO_2_ into a single product, formate. The hematite photoanode achieves continuous and selective oxidation of alkaline pre-treated polyethylene terephthalate (PET) plastics to formate, while an organic semiconductor photocathode coupled to a biocatalyst achieves selective CO_2_ photoreduction to formate under neutral pH conditions. The integrated PEC device operates without external voltage input to achieve simultaneous plastic oxidation and CO_2_ reduction, leading to a near-200% formate Faradaic efficiency and an average formate production rate of 11 μmol cm^−2^ h^−1^ for 10 h under simulated AM1.5G irradiation at room temperature. This work introduces a strategy for the visible-light promoted processing of two distinct waste streams into a single product, thereby enhancing product formation rates, reducing limitations arising from product separation and advancing efforts toward a sustainable circular industry.

## Abstract 2

An organic–inorganic photoelectrochemical (PEC) tandem device for converting polyethylene terephthalate (PET) plastics and carbon dioxide (CO_2_) to formate under simulated solar irradiation.

## Graphical abstract

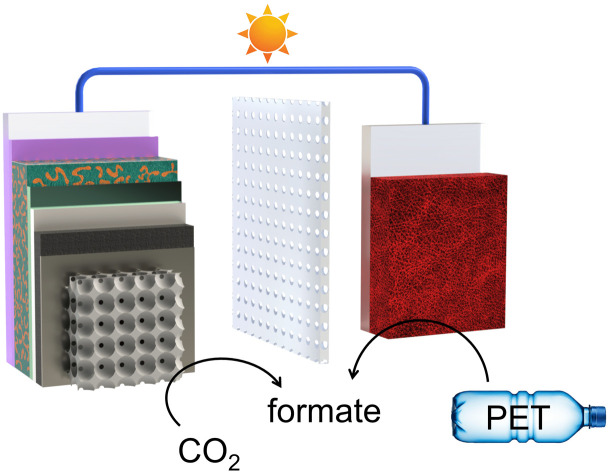

**Broader context**

CO_2_ emissions and PET plastics accumulation have significant environmental impacts and there is an urgent need for innovative methods to produce sustainable platform chemicals. Solar chemical synthesis offers such a transformative approach for storing solar energy in the form of chemical bonds, but photoelectrochemical (PEC) cells commonly only couple fuel production with the challenging water oxidation reaction, which has limited economic value. This work introduces a PEC tandem device constructed from earth-abundant light absorbers and catalysts, featuring a semi-artificial organic photocathode and an all-solution-processed hematite photoanode. The photocathode features a hierarchically structured TiO_2_ layer to host CO_2_ converting enzymes, while the photoanode is homogeneously coated with a nickel-based nanosheet co-catalyst. The device demonstrates the solar comproportionation of PET plastics and CO_2_ for the sustainable production of formate, a versatile chemical feedstock with potential applications in a net zero economy. Operating under one sun irradiation without external voltage input, the integrated device produces formate at both electrodes with high selectivity and photocurrent density. This solar-to-chemical technology converts two abundant waste streams into a clean energy carrier and highlights a promising pathway toward advancing a circular economy.

## Introduction

Rapidly increasing anthropogenic emissions of the greenhouse gas carbon dioxide (CO_2_) and the accumulation of polyethylene terephthalate (PET) plastic waste pose significant environmental challenges, contributing to climate change and persistent plastic pollution.^1,2^ Despite global efforts to mitigate these issues, CO_2_ and PET are not sufficiently appreciated as resources with substantial potential for conversion into valuable chemicals in future circular chemical industries.^3,4^

Photoelectrochemistry employs semiconducting photoelectrodes for solar-driven chemical transformations.^5,6^ Conventional photoelectrochemical (PEC) cells and photovoltaic-electrolysis systems couple water oxidation with CO_2_ reduction or, more recently, PET/ethylene glycol (EG) oxidation with proton reduction, generating molecular hydrogen (H_2_) and oxygen (O_2_) from the counter reactions.^7–9^ Considerable progress has been made in developing PEC devices for CO_2_ photoreduction using photocathode materials such as oxides, chalcogenides, and halide perovskites, enabling the production of useful chemicals such as carbon monoxide (CO), syngas, and formate.^10–13^ Similarly, PEC oxidation of PET and its monomer EG (a primary raw material in PET production and hydrolysis), has demonstrated the potential to produce value-added chemicals such as glycolate and formate.^13,14^ However, these systems face either high thermodynamic requirements (water oxidation) or generate different products in separate compartments, complicating product separation and storage. Consequently, the development of an integrated PEC system capable of coupling PET reforming with CO_2_ photoreduction to produce a single product is both thermodynamically and economically desirable and facilitates product isolation and accumulation.

Formate is a valuable chemical with broad potential applications as a key intermediate in chemical synthesis, a promising energy carrier in fuel cells, a vital metabolite in biological processes (formate bio-refineries),^15^ and is utilised in industries for applications such as solvents, de-icers, and additives.^16,17^ The sustainable production of the ‘hub molecule’ formate could therefore play a crucial role in the development of circular chemistry.

Recent advancements include electrolysers that couple CO_2_ reduction with alcohol oxidation (*e.g.*, methanol,^18^ glycerol,^19^ or EG^20,21^) for sustainable formate synthesis. Solar comproportionation of CO_2_ and soluble biomass molecules to formate has been demonstrated on a floating UV-driven TiO_2_ photocatalyst and a complex dual Si PV-assisted PEC tandem cell.^22,23^ The reported PEC tandem cell utilised two pieces of crystalline Si solar cells to power a pair of Si|GaN photocathode and a hematite photoanode, showing significant device complexity and limited partial current density.^23^ Unbiased PEC systems for EG oxidation coupled with hydrogen evolution reaction (HER) have been recently demonstrated using metal oxide photoelectrodes, but the instability of BiVO_4_ photoanodes under mild alkaline conditions compromises product selectivity.^9^ Recent studies have identified hematite as a promising photoanode material for PET reforming.^24–26^ Despite this progress, the PEC conversion of CO_2_ and PET into a single product has not yet been realised.

In this study, we present an organic–inorganic PEC tandem device for the visible-light-driven comproportionation of CO_2_ and PET to formate. An organic semiconductor (OSC) was selected as the light absorber for fabricating an organic photovoltaic (OPV) photocathode, owing to its distinctive advantages in earth abundance, solution processability, tuneable energy levels, and excellent optoelectronic properties for visible light absorption and charge generation. The photocathode rationally coupled a [W]-formate dehydrogenase (FDH) from *Nitratidesulfovibrio vulgaris* Hildenborough (*Nv*H)§§Formerly known as *Desulfovibrio vulgaris* Hildenborough (*Dv*H). and carbonic anhydrase (CA) from bovine erythrocytes to enable selective and efficient conversion of CO_2_ to formate. For the oxidation of the PET hydrolysate, an all-solution-processed hematite photoanode was employed, chosen for its excellent oxidation performance and stability in strongly alkaline environments. The hematite photoanode was doped with an n-type dopant and uniformly coated with a nickel-based co-catalyst, ensuring selective and durable PET oxidation. The resulting OPV–hematite PEC tandem cell operated under simulated AM1.5G irradiation without external voltage input, achieving simultaneous formate production through both reduction and oxidation reactions.

## Results and discussion

### Overall design of the OPV–hematite tandem device

**Fig. 1:**
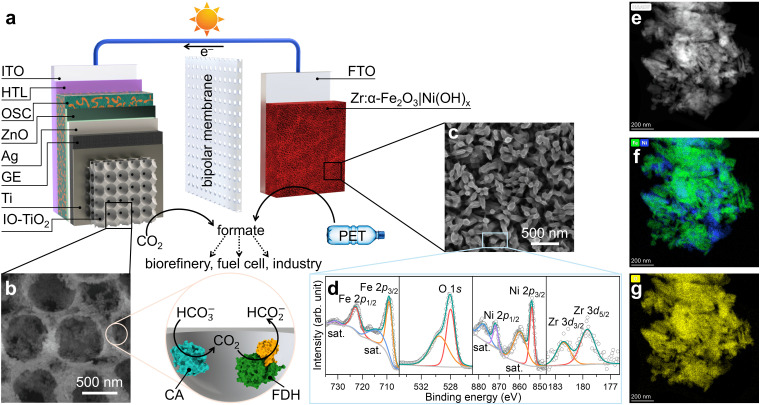
(a) Schematic illustration of the organic–inorganic PEC tandem device. (b) Top-view SEM image of IO-TiO_2_ with illustration of co-immobilisation of FDH (PDB: 6sdv) and CA (PDB: 1v9e). (c) Top-view SEM image, (d) XPS spectra, (e) HAADF image, and TEM elemental mapping for (f) Fe (green), Ni (blue), and (g) O (yellow) of Zr:α-Fe_2_O_3_|Ni(OH)_*x*_.

The organic–inorganic PEC tandem device comprised of an OPV photocathode and a hematite photoanode, with the catholyte and anolyte separated by a bipolar membrane (Fig. 1a and Fig. S1, ESI†). This configuration enabled simultaneous CO_2_ reduction and PET reforming under their separately optimised pH conditions.^27,28^ The OPV semi-artificial photocathode was fabricated following our previous works (see Experimental section for details),^8,29^ where indium tin oxide (ITO) served as the transparent conducting oxide (TCO) substrate. The OSC light absorber was sandwiched between a PEDOT:PSS hole transport layer (HTL) and a zinc oxide (ZnO) electron transport layer (ETL), with silver (Ag) providing the final electrical contact.^8^ The energy level diagram and performance metrics of the 10 OPV devices used in this study can be found in Fig. S2–S4 (ESI†).

The OPV photocathode was encapsulated with graphite epoxy (GE), preventing electrolyte penetration while offering a versatile platform to support various electrocatalysts.^8^ GE was then interfaced with a hierarchically structured inverse-opal titanium dioxide (IO-TiO_2_) overlayer.^30^ This configuration enhanced the surface area and provided a biocompatible metal oxide surface to immobilise FDH and CA in their electroactive orientations, as previously established (Fig. 1b).^29^*Nv*H FDH was selected as the bioelectrocatalyst for CO_2_-to-formate conversion due to its near-unity selectivity across a wide potential range, negligible onset potential, and optimised activity under mild conditions.^31–33^ As the high catalytic turnover frequency of FDH can rapidly deplete local protons, CA was co-immobilised with FDH to avoid a detrimental pH increase in the local environment,^34^ maintaining high CO_2_ reduction performance without the need for non-innocent buffers and additives.^29,35^ The resulting photocathode is denoted as OPV|IO-TiO_2_|FDH+CA.

The hematite photoanode was doped with zirconium (Zr) through hydrothermal synthesis (see Experimental section for details) to enhance the carrier concentration for a higher conductivity.^26^ Mott–Schottky analysis and electrochemical impedance spectroscopy (EIS) reveal that Zr-doped hematite exhibits a higher donor density of 1.88 × 10^19^ cm^−3^ and lower resistivity of 75 kΩ, compared to 1.68 × 10^18^ cm^−3^ and 192 kΩ for undoped hematite (Fig. S5 and S6, ESI†).^26^ Ultraviolet-visible (UV-vis) spectroscopy indicates that Zr doping has minimal impact on the light absorption range (Fig. S7, ESI†).^26^ To improve charge transfer efficiency and product selectivity, a hydrothermally synthesised Ni(OH)_*x*_ overlayer was added as a co-catalyst for the oxidation of PET to formate.^9,24,25^ Scanning electron microscopy (SEM) images of the Zr-doped hematite reveal a worm-like nanowire structure, formed through high-temperature annealing (Fig. S8, ESI†).^36^ This morphology facilitates the diffusion of photogenerated holes to the semiconductor–liquid junction for PEC oxidation reactions. The hydrothermal synthesis of Ni(OH)_*x*_ on hematite (see Experimental section for details) yields a homogeneously coated thin nanosheet overlayer (Fig. 1c and Fig. S8, ESI†).^9,24,25^ X-ray photoelectron spectroscopy (XPS) analysis (Fig. 1d), elemental mapping in transmission electron microscopy (TEM, Fig. 1e–g and Fig. S9, ESI†), and X-ray diffraction (XRD) patterns (Fig. S10, ESI†) confirmed the presence of all intended elements and phase purity, verifying the successful n-type doping of Zr^4+^ and co-catalyst deposition on the Zr:α-Fe_2_O_3_|Ni(OH)_*x*_ photoanode.

### PEC reduction of CO_2_-to-formate using an OPV photocathode

**Fig. 2:**
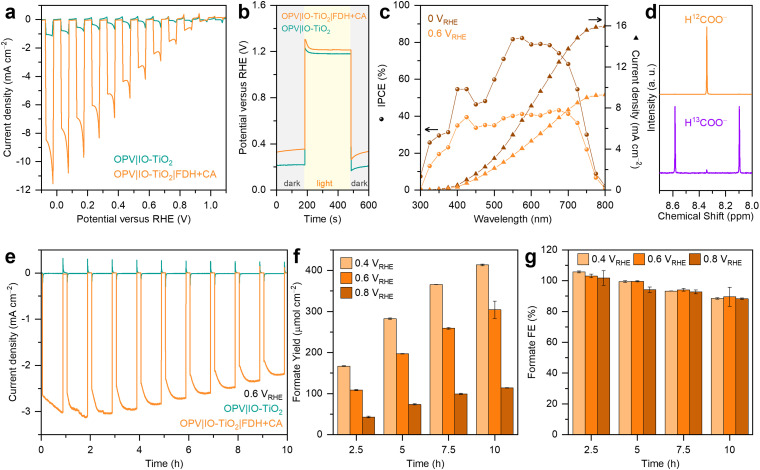
Characterisation of the OPV|IO-TiO_2_|FDH+CA photocathode. (a) Chopped light LSV scans (10 mV s^−1^). (b) Chopped light OCP traces. (c) IPCE and integrated photocurrent densities. (d) ^1^H NMR spectra (400 MHz, D_2_O) of the post 10 h chronoamperometry electrolyte using either ^12^CO_2_/NaH^12^CO_3_ (top) or ^13^CO_2_/NaH^13^CO_3_ (bottom) as the carbon source. (e) Chopped light chronoamperometry traces. (f) Areal formate yield and (g) formate FE as a function of potential. Conditions: simulated AM1.5G irradiation (100 mW cm^−2^), OPV|IO-TiO_2_|FDH+CA photocathode as working electrode (active area = 0.25 cm^2^), stirred 9 mL CO_2_-saturated NaHCO_3_ buffer (50 mM, pH 6.45) containing KCl (50 mM), room temperature.

PEC characterisations of the OPV photocathodes were conducted in a H-type PEC cell using a three-electrode configuration under simulated AM1.5G irradiation from the ITO back-side. The electrolyte contained 9 mL of CO_2_-saturated aqueous NaHCO_3_ buffer (50 mM, pH 6.45) with KCl (50 mM) at room temperature. Linear sweep voltammetry (LSV) of the OPV|IO-TiO_2_|FDH+CA photocathode under chopped AM1.5G irradiation (Fig. 2a) revealed an onset potential of approximately 1.0 V *vs.* the reversible hydrogen electrode (RHE) for CO_2_ reduction (constant light and dark LSV scans in Fig. S11, ESI†), which corresponds well with the expected open circuit potential (OCP) of approximately 1.0 V (Fig. 2b and Fig. S3 and S4, ESI†).^8,29^ The photocathode exhibited a photocurrent density exceeding 10 mA cm^−2^ at 0 V *vs.* RHE, attributed to the synergistic effects of high enzyme loading on a hierarchical support structure,^29,37^ the optimisation of the local environment through the co-immobilisation of CA,^34^ and the robust encapsulation of OPV devices.^8^ Incident photon-to-current conversion efficiency (IPCE) measurements were performed on the OPV|IO-TiO_2_|FDH+CA photocathode at 0 and 0.6 V *vs.* RHE (Fig. 2c), approaching a maximum IPCE of 80% between 550 and 650 nm at 0 V *vs.* RHE. A control experiment using the OPV|IO-TiO_2_ photocathode without enzymes displayed only negligible photocurrent density from the charging of TiO_2_ (Fig. 2a). Isotopic labelling experiments in ^13^CO_2_-saturated NaH^13^CO_3_ electrolyte (50 mM, with 50 mM KCl) confirmed H^13^COO^−^ as the product (^1^H NMR, doublet, coupling constant = 195 Hz; Fig. 2d). Further isotopic labelling experiments using ^13^CO_2_ as the headspace gas in a NaH^12^CO_3_ electrolyte (50 mM, with 50 mM KCl) confirmed that the co-immobilised enzyme CA had converted bicarbonate to dissolved CO_2_ for electrochemical CO_2_-to-formate conversion (Fig. S12, ESI†).

Chronoamperometry of the OPV photocathodes was performed at 0.6 V *vs.* RHE under chopped AM1.5G irradiation for 10 h (Fig. 2e, other potentials can be found in Fig. S13, ESI†) and OPV|IO-TiO_2_|FDH+CA exhibited a stable photocurrent of approximately 3 mA cm^−2^ during the initial 4 h, which gradually declined to around 2.2 mA cm^−2^ after 10 h, with no evidence of significant dark current development. This suggests that the encapsulation remained intact, and the observed decrease in photocurrent is likely due to the gradual inactivation of FDH. For formate quantification, aliquots of the electrolyte were taken every 2.5 h during chronoamperometry at 3 different potentials and subsequently analysed (Fig. 2f and Table S1, ESI†). At 0.6 V *vs.* RHE, the formate yield increased from 109 ± 2 μmol cm^−2^ at 2.5 h to 304 ± 21 μmol cm^−2^ at 10 h, indicating near-linear product accumulation in agreement with the stable photocurrent. At each time interval, the formate yield decreased with more positive potentials due to the dependency of photocurrent on potential (Fig. 2a). The highest formate yield was achieved after 10 h of CO_2_ photoreduction at 0.4 V *vs.* RHE, reaching 413 ± 2 μmol cm^−2^. Based on the formate yield and photocurrent density, the formate Faradaic efficiency (FE) was calculated (Fig. 2g and Table S1, ESI†). Despite differences in photocurrent densities across different potentials, FDH consistently demonstrated high selectivity for CO_2_ reduction to formate for 10 h. At 0.6 V *vs.* RHE, the formate FE values were 103 ± 2%, 99 ± 2%, 94 ± 2%, and 90 ± 6% at 2.5, 5, 7.5, and 10 h, respectively. The headspace gas of the cathodic chamber was analysed using gas chromatography (GC), revealing no detectable reduction by-products such as CO and H_2_ (Fig. S14, ESI†).

### PEC oxidation of PET-to-formate using a hematite photoanode

**Fig. 3:**
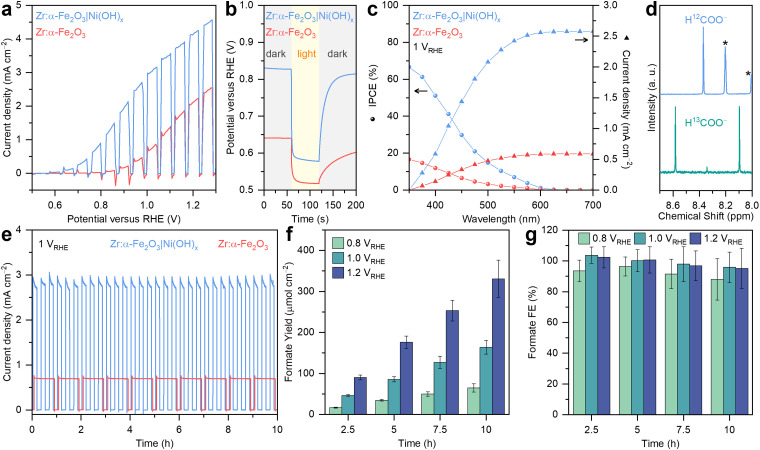
Characterisation of the Zr:α-Fe_2_O_3_|Ni(OH)_*x*_ photoanode. (a) Chopped light LSV scans (10 mV s^−1^). (b) Chopped light OCP traces. (c) IPCE and integrated photocurrent densities. (d) ^1^H NMR spectra (400 MHz, D_2_O) of the post 10 h chronoamperometry electrolyte using either PET hydrolysate (top) or EG-^13^C_2_ (bottom) as the carbon source. Asterisk denotes isophthalate and TPA-^13^C in PET hydrolysate (Fig. S14 and S15, ESI†). (e) Chopped light chronoamperometry traces. (f) Areal formate yield and (g) formate FE as a function of potential. Conditions: simulated AM1.5G irradiation (100 mW cm^−2^), Zr:α-Fe_2_O_3_|Ni(OH)_*x*_ photoanode as working electrode (active area = 0.15 cm^2^), stirred 9 mL N_2_-saturated PET hydrolysate (0.1 g mL^−1^) in KOH (1 M, pH ~14) at room temperature.

The oxidative reforming of PET plastics into useful chemicals was carried out using a two-step process. In the first step, a real-world PET bottle with a crystallinity of 24% (Fig. S15 and S16, ESI†) were shredded into small pieces and subjected to alkaline hydrolysis in 1 M aqueous KOH (see Experimental section for details) to convert them into soluble EG and terephthalate (TPA, Fig. S17, ESI†). In the second step, the PET hydrolysate was purged with N_2_ and directly used as the electrolyte for PEC measurements on the all-solution-processed hematite photoanodes. Chopped light LSV (Fig. 3a) of Zr:α-Fe_2_O_3_ revealed an onset potential at around 0.75 V *vs.* RHE and a photocurrent density approaching 2.1 mA cm^−2^ at 1.2 V *vs.* RHE (constant light and dark LSV scans in Fig. S18, ESI†). Recent studies have highlighted the superior catalytic activity of Ni-based co-catalysts, often in nanoparticle form, for PET oxidation.^24,25^ However, achieving a homogeneous coating and integrating these materials into a PEC tandem device remain significant challenges due to difficulties in device design, competing reactions, and selection of semiconductors.^38,39^

Upon modification of Zr:α-Fe_2_O_3_ with a uniformly coated Ni(OH)_*x*_ nanosheet overlayer, a 150 mV cathodic shift in onset potential and a twofold increase in photocurrent density at 1.2 V *vs.* RHE were observed (Fig. 3a), demonstrating the effectiveness of Ni(OH)_*x*_ as a co-catalyst for PET reforming.^9,24,25^ As shown in Fig. 3b, Zr:α-Fe_2_O_3_ and Zr:α-Fe_2_O_3_|Ni(OH)_*x*_ exhibited dark OCP of 0.64 and 0.83 V *vs.* RHE, respectively, with corresponding photovoltages of 0.25 and 0.12 V. These differences suggest that Fermi level pinning to surface states occurs on the pristine hematite photoanode,^40^ while co-catalyst deposition can mitigate the level of pinning.^41^ Moreover, when illumination was ceased at 120 s, the OCP of the Ni(OH)_*x*_-modified hematite photoanode equilibrated to the dark condition significantly faster than the pristine one. This indicates that Ni(OH)_*x*_ effectively suppressed surface recombination,^42^ enhancing the charge transfer efficiency during pre-treated PET reforming.

To assess the impact of Ni(OH)_*x*_ on quantum efficiency, IPCE measurements were performed at 1.0 V *vs.* RHE for both Zr:α-Fe_2_O_3_ and Zr:α-Fe_2_O_3_|Ni(OH)_*x*_ (Fig. 3c). Both IPCE spectra exhibited an onset at around 600 nm, consistent with the bandgap of hematite. The IPCE increased with higher photon energy (shorter wavelength), a characteristic behaviour of inorganic semiconductors. Upon the introduction of Ni(OH)_*x*_, the maximum IPCE at 350 nm significantly increased from 17% to 67%, highlighting the substantial improvement in charge transfer and extraction facilitated by Ni(OH)_*x*_. The integrated photocurrent densities at 1.0 V *vs.* RHE were calculated as 0.6 and 2.6 mA cm^−2^ for Zr:α-Fe_2_O_3_ and Zr:α-Fe_2_O_3_|Ni(OH)_*x*_, respectively. These values differ slightly from those obtained in the LSV scans (Fig. 3a), a common observation for metal oxide electrodes. This discrepancy arises from non-linear recombination effects influenced by factors such as surface states and ferroic behaviours under varying light intensities.^43,44^

To identify the oxidation products of PET, ^1^H NMR analysis was conducted on the PET hydrolysate after PEC reforming (Fig. 3d). A formate peak at 8.37 ppm was observed, alongside another peak at 8.20 ppm (isophthalate, Fig. S19–S21, ESI†) and TPA below 8.0 ppm (Fig. S19, ESI†).^26^

Recent spectroscopic studies revealed that the Ni(OH)_*x*_ catalyst is advantageous for the selective conversion of EG oxidation intermediates into formate in alkaline conditions.^9,38^ In brief, glycolaldehyde, the first oxidation intermediate, is rapidly converted to glyoxal and glycolate, which then undergo oxidative C–C bond cleavage to produce formate (Fig. S22, ESI†). To further verify the carbon source of formate formation, an isotopic labelling experiment was performed using EG-^13^C_2_ (0.1 M) in KOH (1 M, pH 14) as the electrolyte. Following PEC oxidation of EG-^13^C_2_, a doublet ^13^C-formate signal with a coupling constant of 195 Hz appeared in the ^1^H NMR spectrum (Fig. 3d), conclusively demonstrating that formate originated from EG oxidation in the PET hydrolysate.

The stability of the hematite photoanode for PEC reforming of the PET hydrolysate was evaluated using chronoamperometry at 1.0 V *vs.* RHE (Fig. 3e, other potentials can be found in Fig. S23, ESI†). The Zr:α-Fe_2_O_3_|Ni(OH)_*x*_ photoanode demonstrated a stable photocurrent of 3.0 mA cm^−2^, significantly higher than the 0.7 mA cm^−2^ observed for the pristine Zr:α-Fe_2_O_3_ photoanode. The formate yield during chronoamperometry on Zr:α-Fe_2_O_3_|Ni(OH)_*x*_ photoanodes was quantified at 2.5 h intervals (Fig. 3f, Fig. S24, and Table S2, ESI†), revealing an expected potential dependency, where higher potentials resulted in greater formate production, consistent with the photocurrent trend observed in the LSV scans (Fig. 3a). In contrast, PET oxidation on a pristine Zr:α-Fe_2_O_3_ photoanode results in a range of oxidation products such as formate, glycolate, and acetate (Fig. S25, ESI†), similar to the observations reported by Park and colleagues.^26^ At 1.0 V *vs.* RHE, the formate yield increased nearly linearly from 46.3 ± 2.3 μmol cm^−2^ at 2.5 h to 163.9 ± 16.8 μmol cm^−2^ at 10 h, indicating a stable partial current density for PET oxidation to formate (Fig. 3f). The highest formate yield, 330.9 ± 45.45 μmol cm^−2^, was achieved at 1.2 V *vs.* RHE after 10 h of chronoamperometry. The formate FE at 1.0 V *vs.* RHE was determined to be 104 ± 5%, 100 ± 7%, 98 ± 11%, and 96 ± 10% at 2.5, 5, 7.5, and 10 h, respectively (Fig. 3g and Table S2, ESI†).

The near-unity selectivity confirms the excellent activity and stability of the Zr:α-Fe_2_O_3_|Ni(OH)_*x*_ photoanode for the alkaline oxidation of PET hydrolysate to formate. It also inspires future research into developing photoanodes and co-catalysts with enhanced stability under strongly alkaline and high-temperature conditions, enabling the direct utilisation of untreated PET.

### PEC comproportionation of PET and CO_2_ to formate

**Fig. 4:**
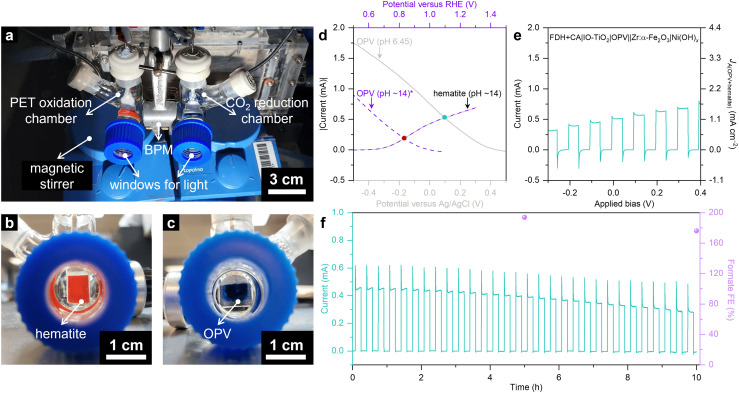
PEC tandem cell. (a) Photograph of the 2-electrode PEC cell. BPM refers to bipolar membrane. (b) Photograph of the PET oxidation chamber with a hematite photoanode (active area = 0.2 cm^2^). (c) Photograph of the CO_2_ reduction chamber with an OPV photocathode (active area = 0.25 cm^2^). (d) Anticipation of photocurrent overlap (green dot) in 2-electrode photoelectrolysis cell at 0 V applied voltage based on the 3-electrode studies of the individual photoelectrodes (two solid grey traces; plotted *versus* Ag/AgCl reference electrode). The pH difference in the 2-electrode photoelectrolysis cell contributes a chemical bias. The estimate of a hypothetical current without chemical bias (ΔpH) is shown by the crossover (red dot) of the hematite photoanode and the OPV photocathode (two purple dashed traces; plotted *versus* RHE reference electrode). The asterisk denotes the imaginary OPV (purple dashed) trace with theoretical adjustment to pH ~14 to subtract the chemical bias. The sign of the OPV photocathodic trace is reversed to improve visualisation of the current overlap. (e) LSV scan of a FDH+CA|IO-TiO_2_|OPV‖Zr:α-Fe_2_O_3_|Ni(OH)_*x*_ PEC tandem device under 2-electrode configuration. (f) Chopped photoelectrolysis trace of the PEC tandem device under 0 V applied voltage. Conditions: simulated AM1.5G irradiation (100 mW cm^−2^), catholyte contains stirred 9 mL CO_2_-saturated NaHCO_3_ buffer (50 mM) and KCl (50 mM) at room temperature (pH 6.45), anolyte contains stirred 9 mL N_2_-saturated PET hydrolysate (0.1 g mL^−1^) in KOH (1 M, pH ~14, room temperature).

An OPV–hematite PEC tandem device was constructed by coupling the OPV|IO-TiO_2_|FDH+CA photocathode with the Zr:α-Fe_2_O_3_|Ni(OH)_*x*_ photoanode in a two-compartment H-type cell for PEC comproportionation of PET and CO_2_ into formate (Fig. 4a). The anolyte contained a N_2_-purged PET hydrolysate with EG and TPA in KOH (1 M, pH 14, Fig. 4b), while the catholyte contained a CO_2_-saturated NaHCO_3_ buffer (50 mM, pH 6.45) with KCl (50 mM, Fig. 4c). A bipolar membrane separated the two compartments, maintaining the pH gradient to operate both reduction and oxidation reactions at their optimised conditions. Both photoelectrodes were irradiated simultaneously in a side-by-side configuration.

Previous studies on solar water splitting cells have demonstrated that water dissociation at the bipolar membrane does not alter the overall cell bias, as the redox potential shifts equally for both half-reactions.^27,28,45^ In contrast, recent work on non-water splitting cells has shown that the pH difference induces an internal chemical bias (ΔpH) following Nernstian behaviour (∼440 mV for ∼7.5 pH difference).^13^ To anticipate the photocurrent overlap at 0 V applied bias, this internal chemical bias was accounted for by plotting the photocurrent of both electrodes obtained from the respective 3-electrode analysis against the Ag/AgCl scale, yielding a photocurrent of 0.52 mA at ∼0.1 V *vs.* Ag/AgCl (Fig. 4d). LSV in a two-electrode configuration using the tandem PEC cell revealed a rising photocurrent during the forward scan (Fig. 4e), with a photocurrent of 0.51 mA observed at 0 V applied bias with internal chemical bias from the pH difference in the two compartments.

Chopped photoelectrolysis at 0 V applied voltage showed large transient photocurrent spikes (Fig. 4f), which are likely attributable to recombination losses at the surface of the photoelectrodes.^13^ The steady-state photocurrent gradually decreased from 0.45 to 0.28 mA over 10 h, with an average stable photocurrent at around 0.37 mA (Fig. 4f). The photocurrent decay likely originated from the gradual deactivation of FDH, as indicated by similar chronoamperometry trends at the photocathode (Fig. 2e). Moreover, no significant dark current development was observed, suggesting that the OSC light absorber and encapsulated device components remained intact.

The comproportionation of pre-treated PET and CO_2_ in the PEC tandem cell achieved a remarkable formate FE of 194% (cathodic compartment: 97%, anodic compartment: 97%) and 176% (cathodic compartment: 92%, anodic compartment: 84%) after 5 and 10 h, respectively. This highlights the unique advantage of coupling two solar reforming processes to selectively produce a single product with near 200% FE. Compared with state-of-the-art PEC tandem devices for direct solar formate synthesis (Table S3, ESI†),^9,13,23,37,46–48^ the FDH+CA|IO-TiO_2_|OPV‖Zr:α-Fe_2_O_3_|Ni(OH)_*x*_ device demonstrated outstanding performance, achieving a formate production rate of 11 μmol cm^−2^ h^−1^, and a formate FE of 176%. The apparent quantum efficiency (AQE) of the PEC tandem device is estimated to be 5.8%, comparable to other unbiased solar-driven formate synthesis systems such as 10.4% for Bi|GaN|Si‖2 × Si-PV‖Ti:α-Fe_2_O_3_|NiOOH,^23^ 2.7% for FDH|IO-TiO_2_|PVK‖NiF|Cu_27_Pd_73_,^13^ and 1.6% for RuO_*x*_|Cu_2_O‖Mo:BiVO_4_|NiCo-LDH.^9^ These results confirm the potential of this PEC tandem cell design for enabling PEC comproportionation of various reactions, paving the way for future innovations in solar-driven chemical transformations. The co-production of formate in aqueous solution not only enables its direct utilisation for microbial cascade conversion in formate bio-refineries in future applications but also highlights the need for further studies on product separation, exploring emerging techniques such as ion exchange and membrane filtration.

## Conclusions

This study introduces a standalone organic–inorganic PEC tandem device capable of simultaneously utilising CO_2_ and PET for sustainable formate production. The OPV photocathode achieved a benchmark photocurrent exceeding 10 mA cm^−2^ at 0 V *vs.* RHE, demonstrating near-unity selectivity for CO_2_-to-formate conversion across various potentials when paired with FDH. Meanwhile, the hematite photoanode, enhanced through rational morphology control, n-type doping, and co-catalyst deposition, exhibited a photocurrent density surpassing 4 mA cm^−2^ at 1.2 V *vs.* RHE using PET hydrolysate as the electrolyte. This enabled selective oxidation of the soluble PET component (EG) to formate with nearly 100% FE. The two-compartment PEC tandem cell operated efficiently at 0 V voltage bias, achieving simultaneous CO_2_ photoreduction and PET photooxidation at an average stable photocurrent of 0.37 mA over 10 h. This integrated device yielded a near-200% formate FE and a formate production rate of 11 μmol cm^−2^ h^−1^. Unlike conventional PEC devices focused on either water splitting or CO_2_ reduction, this system couples the utilisation of both waste streams for clean formate synthesis. This work highlights a transformative approach in solar energy conversion, offering a sustainable pathway to simultaneously upcycle greenhouse gas CO_2_ and waste PET plastics into a single product.

## Experimental section

### Materials

The chemicals and materials were purchased from commercial suppliers and used without further purification: N_2_ and CO_2_ gas bottles (2% CH_4_ as internal standard, BOC), carbon–^13^C dioxide (^13^CO_2_, Sigma-Aldrich, 99.0 atom% ^13^C), iron(iii) chloride hexahydrate (FeCl_3_·6H_2_O, Sigma-Aldrich, ≥98%), sodium nitrate (NaNO_3_, Thermo Scientific Chemicals, 99.0% min), zirconium(iv) chloride (ZrCl_4_, Thermo Scientific Chemicals, 99.5+%), nickel(ii) nitrate hexahydrate (Ni(NO_3_)_2_·6H_2_O, Thermo Scientific Chemicals, 99%), ammonium fluoride (NH_4_F, Sigma-Aldrich, ≥99.99%), urea (CH_4_N_2_O, Chem-Lab NV, 99.5–100.5%), ethylene glycol-^13^C_2_ (HO^13^CH_2_^13^CH_2_OH, Sigma-Aldrich, 99 atom% ^13^C), potassium hydroxide (KOH, Fisher Chemical, Analytical Reagent grade), isophthalic acid (C_8_H_6_O_4_, Thermo Scientific Chemicals, 99%), titanium dioxide nanoparticles (Aeroxide TiO_2_ P25, Evonik Industries, 21 nm diameter), polystyrene beads (750 nm diameter, Polysciences Inc., 2.7% w/v suspension in water), titanium foil (0.25 mm thick, Alfa Aesar, 99.5%), Zn (dust, ACROS, 98+%), hydrochloric acid (HCl, Honeywell Fluka, 37%), poly(3,4-ethylenedioxythiophene)–poly(styrenesulfonate) (PEDOT:PSS, Clevios P VP AI 4083, Heraeus), poly[4,8-bis(5-(2-ethylhexyl)thiophen-2-yl)benzo[1,2-*b*;4,5-*b*′]dithiophene-2,6-diyl-*alt*-(4-(2-ethylhexyl)-3-fluorothieno[3,4-*b*]thiophene-)-2-carboxylate-2-6-diyl] (PCE10, 1-material), 5,5′-[[4,4,9,9-tetrakis(2-ethylhexyl)-4,9-dihydro-*s*-indaceno[1,2-*b*:5,6-*b*′]dithiophene-2,7-diyl]bis(2,1,3-benzothiadiazole-7,4-diylmethylidyne)]bis[3-ethyl-2-thioxo-4-thiazolidinone] (EH-IDTBR, 1-material), zinc oxide nanoparticles (−3.9 eV work function, Avantama), chlorobenzene (extra dry over molecular sieves ≥99.5%, ACROS), graphite powder (<20 μm, synthetic, Sigma-Aldrich), araldite standard 2-part epoxy, araldite 5-minute rapid 2-part epoxy, DL-dithiothreitol (DTT, Sigma-Aldrich, >99.5%), 2-amino-2-(hydroxymethyl)-1,3-propanediol (Tris base, Sigma-Aldrich, ≥99.8%), carbonic anhydrase from bovine erythrocytes (Sigma-Aldrich, ≥95%, specific activity ≥3500 W-A units mg^−1^ protein, lyophilised powder), sodium bicarbonate (NaHCO_3_, Sigma-Aldrich, ≥99.9%), sodium bicarbonate-^13^C (NaH^13^CO_3_, Sigma-Aldrich, 98 atom% ^13^C), potassium chloride (KCl, ACROS, 99.999%), water (H_2_O, Fisher Chemical, HPLC Gradient grade), sulfuric acid solution (H_2_SO_4_, Honeywell Fluka, for HPLC 49–51%), ethanol (C_2_H_5_OH, Sigma-Aldrich, 96%), deuterium oxide (D_2_O, Sigma-Aldrich, 99.9 atom% D, contains 0.75 wt% 3-(trimethylsilyl) propionic-2,2,3,3-*d*_4_ acid sodium salt), fluorine doped tin oxide (FTO) coated glass slide (Solaronix), parafilm (Bemis), and rubber septa (Subaseal). Unless stated otherwise, MilliQ H_2_O (18.2 MΩ cm) was used for all the experiments except for HPLC. [W]-FDH from *Nitratidesulfovibrio vulgaris* Hildenborough (*Nv*H) was expressed and purified according to previously reported methods.^31,49^

### Fabrication of Zr:α-Fe_2_O_3_|Ni(OH)_*x*_ photoanodes

Zr:α-Fe_2_O_3_ photoanodes were synthesised by a hydrothermal-annealing method at optimised conditions as previously reported.^26,36,42^ Chemical bath deposition of Zr:β-FeOOH onto a FTO coated glass substrate was conducted in a precursor solution containing FeCl_3_ (0.15 M), NaNO_3_ (1 M), and ZrCl_4_ (2 mM) at 100 °C for 3 h. The Zr:β-FeOOH thin film was annealed at 800 °C for 15 min to form Zr:α-Fe_2_O_3_ nanostructures. The hydrothermal deposition of Ni(OH)_*x*_ co-catalyst was carried out by soaking a hematite photoanode into a precursor solution containing Ni(NO_3_)_2_ (10 mM), NH_4_F (20 mM), and urea (40 mM) at 110 °C for 2 h.^9,24,25^

### Alkaline hydrolysis of real-world PET bottles

After the removal of labels and caps, the plastic bottle was cut into small pieces and flash frozen in liquid nitrogen.^13^ The size of these PET pieces was further reduced by a grinder before added into KOH (1 M) at a concentration of 0.1 g mL^−1^. The depolymerisation process was conducted by heating the PET suspension at 80 °C for 120 h under stirring. The resulting PET hydrolysate contains EG (0.32 M) and TPA (0.32 M) in aqueous KOH (1 M, pH 14, Fig. S17, ESI†). The PET concentration (10–100 mg mL^−1^) has only a minor effect on hematite photoanode performance, with higher PET concentrations leading to slightly increased photocurrent (Fig. S26, ESI†).

### Differential scanning calorimetry (DSC)

DSC measurements were conducted using a Mettler Toledo differential scanning calorimeter (TGA/DSC 2 STAR^e^ System) equipped with a Mettler Toledo GC100 gas controller. The PET sample was heated at a rate of 10 °C min^−1^ from ambient temperature to 300 °C under a N_2_ atmosphere. The crystallinity was determined from the DSC curve (Fig. S16, ESI†).

### Fabrication of inverse opal TiO_2_ (IO-TiO_2_) electrodes

IO-TiO_2_ electrodes (geometrical surface area = 0.19 cm^2^) with thick films (~37 μm) on Ti foil (1 × 2 cm^2^) were prepared based on our previous work.^29^ Briefly, TiO_2_ nanoparticles (30 mg) were sonicated in a water/methanol mixture (300 μL, 4 : 1 volume ratio). Polystyrene beads (1 mL) were centrifuged at 10 000 rpm and supernatant removed, followed by further washing with MeOH. The TiO_2_ nanoparticle suspension (300 μL) was then added to the polystyrene bead pellet and sonicated for 5 min (<5 °C). The dispersion was drop-cast onto the Ti foil electrodes and annealing was performed in a furnace at 500 °C for 20 min (1 °C min^−1^ ramp rate).

### Fabrication of OPV devices

Conventional structure OPVs of the blend PCE10:EH-IDTBR were fabricated based on our previous works.^8,29^ Washed and patterned ITO-glass substrates (1.3 × 1.3 cm^2^) were subjected to UV-ozone treatment for 40 min, yielding a hydrophilic surface for the facile spin-coating of the PEDOT:PSS (4000 rpm), which was followed by annealing in air at 383 K for 40 min. The active layer PCE10:EH-IDTBR was prepared in chlorobenzene (1 : 2 weight ratio, 24 mg mL^−1^) and spin-coated at 3000 rpm. ZnO nanoparticles were then spin coated at 4000 rpm as the electron-transport layer. Finally, Ag (100 nm) was thermally evaporated, defining an active area of ∼0.5 × 0.5 cm^2^.

### Characterisation of OPV devices

The performance of all OPVs were measured using a Sun 2000 Solar Simulator (Abet Technologies) at room temperature. A certified RS-OD4 reference silicon diode was used to calibrate the light source for 1 sun illumination (AM1.5G, 100 mW cm^−2^). No additional masking of the OPV was required. *J*–*V* scans between −0.1 V and 1.1 V in the reverse and forward direction were collected at a scan rate of 100 mV s^−1^ over 20 mV steps (Keithley 2635 source meter). The shutter was switched on for dark *J*–*V* measurements. The active area of each device (∼0.5 × 0.5 cm^2^) was measured manually.

### Fabrication of OPV photocathodes

Araldite standard 2-part epoxy and graphite powder were thoroughly mixed in a 4 : 3 weight ratio to obtain fresh graphite epoxy (GE) paste. The GE paste was then doctor-bladed onto the OPV and the Ti foil|IO-TiO_2_ electrode firmly pressed onto the paste directly for strong adhesion. A copper wire was attached to the electrode, followed by encapsulation of the entire device with araldite 5-minute rapid 2-part epoxy to protect moisture-sensitive OPV components.

### Enzyme preparation for OPV|IO-TiO_2_|FDH+CA photocathodes

Stock solutions of FDH (50 μM in 20 mM TRIS-HCl, 10% glycerol, 10 mM NaNO_3_, pH 7.6) were stored at −40 °C in an anaerobic glovebox. Prior to each experiment, FDH (10 μL of 50 μM FDH stock) was thawed, incubated and activated with DTT (in 20 mM TRIS-HCl buffer, pH 9) for 20 min.^32^ The enzyme loading was varied and optimised to achieve high current density while maintaining near-unity selectivity. A high FDH loading of 500 pmol provided optimal activity, while a CA loading of 100 pmol effectively regulated the local pH environment without compromising selectivity.^29^

### Mott–Schottky analysis

Electrochemical impedance spectroscopy (EIS) measurements of the hematite electrodes were conducted using a BioLogic VSP potentiostat over a frequency range of 0.5 MHz to 0.1 Hz with a sinusoidal amplitude of 25 mV under dark conditions. The hematite electrode was employed as the working electrode in a single-compartment electrochemical cell, with KOH (1 M, pH ~14) as the electrolyte, a platinum mesh as the counter electrode, and a RE-61AP Hg/HgO reference electrode. Impedance data was fitted to a Randles circuit model using ZView2 software (Scribner Associates). Mott–Schottky plots were derived from the capacitance values obtained, following the Mott–Schottky equation. A surface roughness factor of 20 was incorporated into the EIS analysis to account for the nanostructured nature of the working electrode.^50^

### Photoelectrochemical measurements

All PEC measurements were carried out under simulated AM1.5G conditions (100 mW cm^−2^) using either a Newport Oriel 67005 solar light simulator for 3-electrode configuration or a LOT-quantum design solar light simulator for 2-electrode configuration measurements. Calibration was performed using a certified Newport 843-R optical power meter. Experimentally measured potentials against reference electrodes were converted to the RHE scale using the Nernst equation, for CO_2_ reduction this is a rough estimate.^51^ The PEC CO_2_ reduction experiments were conducted in a 2-compartment electrochemical cell with a 3-elecrode configuration: an OPV|IO-TiO_2_ working electrode, a Pt mesh counter electrode, a RE-6 Ag/AgCl reference electrode, and a Nafion ion exchange membrane. The CO_2_-saturated electrolyte contained KCl (50 mM) and NaHCO_3_ (50 mM, pH 6.45). The PET photooxidation experiments were conducted in a 2-compartment electrochemical cell with a 3-electrode configuration: a hematite working electrode, a Pt mesh counter electrode, a RE-61AP Hg/HgO reference electrode, and a Nafion ion exchange membrane. The N_2_-saturated electrolyte is PET hydrolysate in KOH (1 M, pH ~14) from the alkaline hydrolysis of real-world PET bottles.

The PEC tandem cell is constructed in a 2-compartment electrochemical cell with an OPV|IO-TiO_2_ and a hematite as the working and counter electrodes, separated by a bipolar membrane. The anolyte is N_2_-saturated PET hydrolysate (pH 14) and the catholyte is CO_2_-saturated NaHCO_3_ buffer (50 mM, pH 6.45) containing KCl (50 mM). The photocurrent density (*J*) is defined as *J* = *I*/*A*, where *I* is the current and *A* is the photoactive area. In a 2-electrode configuration, *A* is the sum of the photoactive areas of both photoelectrodes.

IPCE measurements were conducted using a monochromator coupled to a 300 W Xe light source (LOT-Quantum Design MSH-300) and an Ivium CompactStat potentiostat. The light intensity at each wavelength was measured using a Thorlabs PM100D power meter connected to a Thorlabs S302C thermal power sensor. The wavelength (full-width at half-maximum of 15 nm) was increased in 25 nm steps from 300 nm to 800 nm every 30 s. EQE was calculated using the equation: IPCE (%) = *hcJ*/(*eλP*_*λ*_) × 100, where *h* is the Planck constant, *c* is the speed of light, *J* is the photocurrent density, *e* is the elementary charge, *λ* is the wavelength and *P*_*λ*_ is the wavelength-dependant light intensity flux.

### Product quantification

Formate production was monitored *via* quantitative ^1^H NMR spectroscopy (Fig. S27, ESI†) and high-performance liquid chromatography (HPLC, Fig. S24, ESI†). Aliquots of the electrolyte were diluted in D_2_O containing 0.75 wt% 3-(trimethylsilyl)propionic-2,2,3,3-*d*_4_ acid sodium salt as an internal standard and measured by ^1^H NMR spectroscopy (400 MHz, time delay = 60 s). Integration of the NMR spectra were performed using the software MestReNova. A Waters HPLC system with a Phenomenex Rezex ROA-Organic acid H^+^ (8%) column at a column temperature of 75 °C was used. The samples were analysed in the isocratic flow mode (Waters 1525, flow rate: 0.5 mL min^−1^, 5 mM aqueous H_2_SO_4_) using a Waters breeze system equipped with a refractive index detector (Waters 2414) and a diode array UV-vis (*λ* = 210 nm) detector (Waters 2489). Aliquots of the anolyte were removed from the sealed anodic chamber, neutralised with H_2_SO_4_ and centrifuged before placing the supernatant into the autosampler (Waters 2707). The Faradaic efficiency (FE) was calculated using the equation: FE (%) = *nZF*/*Q* × 100, where *n* is the number of moles of formate produced, *Z* is the number of electrons needed per molecule of product (*Z* = 2 for CO_2_-to-formate and *Z* = 3 for EG-to-formate), *F* is the Faraday constant (96 485 C mol^−1^), and *Q* is the total charge passed. The total charge *Q* was determined by integrating the current trace over a defined period.

The FE for comproportionation of CO_2_ and PET to formate was calculated by:
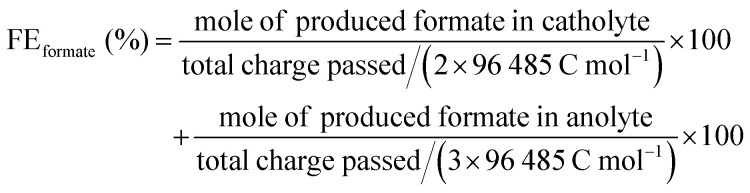


Headspace gas analysis was performed using a Shimadzu Tracera GC-2010 Plus equipped with a barrier discharge ionisation detector. The system featured a ShinCarbon micro ST column (0.53 mm diameter) maintained at 40 °C with helium as the carrier gas. Aliquots of the headspace gas (100 μL) were extracted from the sealed cathodic chamber using a gastight Hamilton syringe. Methane was used as an internal standard.

### Apparent quantum efficiency (AQE)

AQE for the PEC tandem cell was calculated by:
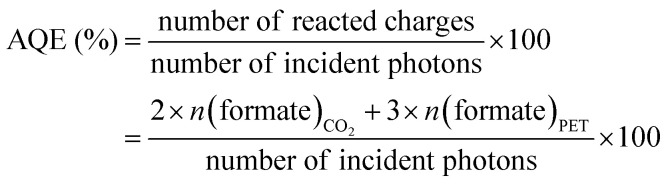
where *n*(formate) is number of produced formate molecules in the corresponding half-reaction, with each formate molecule requiring 2 reacted electrons from CO_2_ reduction and 3 reacted electrons from EG oxidation, the number of incident photons was estimated by integrating the visible light range of the reference AM1.5G spectrum.

### Materials characterisations

Field emission scanning electron microscope images were recorded on TESCAN FEG-SEM instruments (MIRA3 for IO-TiO_2_ and CLARA 2 for hematite), both at an accelerating voltage of 5 kV (in-beam secondary electron detector for IO-TiO_2_; in-column axial SE detector and in-column energy-filtered multidetector for hematite). Transmission electron microscopy imaging and high-angle annular dark-field scanning transmission electron microscopy (HAADF-STEM) imaging were conducted on a Thermo Scientific Talos F200X G2 TEM operating at 200 kV. XPS data were acquired on a Thermo Scientific Escalab 250Xi fitted with a monochromated aluminum Kα X-ray source (1486.7 eV) at a pressure below 10^−8^ Torr and a room temperature of 294 K. X-ray diffraction (XRD) data were collected on a Panalytical Empyrean XRD equipment (Cu Kα radiation), varying the incident beam angle between 5.0° and 90° with a step size of 0.01°. UV-Vis spectra were collected using a Cary 60 UV-vis spectrometer.

## Author contributions

Yongpeng Liu conceptualisation, data curation, formal analysis, funding acquisition, investigation, methodology, project administration, software, resources, visualisation, writing – original draft, writing – review & editing; Celine Wing See Yeung data curation, formal analysis, investigation, methodology, software, resources, visualisation, writing – review & editing; Erwin Reisner funding acquisition, project administration, resources, supervision, validation, writing – original draft, writing – review & editing.

## Conflicts of interest

There are no conflicts to declare.

## Supplementary Material

EE-018-D5EE00689A-s001

## Data Availability

The data that support the findings of this study are available from the University of Cambridge data repository: https://doi.org/10.17863/CAM.117121.
