# Supplementary material for: Photoelectrochemical comproportionation of pre-treated PET plastics and CO2 to formate
Source: Energy Environ Sci. 2025 Apr 11;18(14):7023–33. doi: 10.1039/d5ee00689a (PMC11986568; doi:10.1039/d5ee00689a)
Supplement: EE-018-D5EE00689A-s001 [file EE-018-D5EE00689A-s001.pdf]

Supplementary Information (SI) for Energy & Environmental Science.
This journal is © The Royal Society of Chemistry 2025

Energy & Environmental Science

Electronic Supplementary Information (ESI)

Photoelectrochemical comproportionation of pre-treated PET plastics and CO_2 to formate

Yongpeng Liu, ‡ Celine Wing See Yeung ‡ and Erwin Reisner *

Yusuf Hamied Department of Chemistry, University of Cambridge, Cambridge CB2 1EW, UK. E-mail: reisner@ch.cam.ac.uk

‡ These authors contributed equally to this work.

Cite this: DOI: 10.1039/d5ee00689a

Energy & Environmental Science

Contents

S1 Photographs of Photoelectrodes S6

S2 Energy Level Diagram of All Layers in the OPV S7

S3 OPV Performance Metrics S8

S4 Champion OPV Device S9

S5 Mott−Schottky Analysis of Hematite S10

S6 Nyquist Plot of Hematite S11

S7 Ultraviolet–Visible (UV–Vis) Spectroscopy of Hematite S12

S8 SEM Images of Hematite S13

S9 TEM Images and Elemental Mapping of Hematite S13

S10 XRD Patterns of Hematite S14

S11 Linear Sweep Voltammetry of OPV Photocathodes S15

S12 Isotopic Labelling Experiment Using $^{13}CO_2$/$NaH^{12}CO_3$ S16

S13 Chronoamperometry of OPV Photocathodes S17

S14 Gas Chromatography (GC) of the Cathodic Chamber S18

S15 A Real-World PET Bottle That Was Used for Alkaline Hydrolysis S19

S16 Differential Scanning Calorimetry (DSC) S20

S17 Quantitative ^{1}H NMR Spectrum of PET Hydrolysate S21

S18 Linear Sweep Voltammetry of Hematite Photoanodes S22

S19 Zoomed in ^{1}H NMR Spectra of PET Hydrolysate S23

S20 ^{1}H NMR Spectrum of Isophthalate S24

S21 ^{1}H NMR Spectra of Formate in PET Hydrolysate S25

S22 Proposed Reaction Pathway for EG Oxidation to Formate S26

S23 Chronoamperometry of Hematite Photoanodes S26

S24 HPLC Chromatograms S27

S25 Zoomed in ^{1}H NMR spectrum of Post-PEC Electrolyte S28
S26 Linear Sweep Voltammetry of Hematite Photoanodes with Different Concentrations of PET Hydrolysate. S29
S27 Quantitative ^{1}H NMR Spectrum of Post-PEC Catholyte. S30
S28 Numerical Data for CO_2 Photoreduction on OPV Photocathodes S31
S29 Numerical Data for PET Reforming on Hematite Photocathodes S32
S30 Comparison among State-of-the-art PEC Tandem Cells for Solar Formate Production. S33
References S34

List of Figures

S1 Photographs of photoelectrodes S6

S2 Energy level diagram of all layers in the OPV S7

S3 Histograms of the photovoltaic performance metrics S8

S4 Forward and reverse J-V scans of the champion OPV device S9

S5 Mott−Schottky plots of hematite photoanodes S10

S6 Nyquist plot of hematite S11

S7 UV–Vis spectra of hematite photoanodes S12

S8 SEM images of hematite S13

S9 TEM images and elemental mapping of hematite S13

S10 XRD patterns of hematite S14

S11 Linear sweep voltammetry of OPV photocathodes S15

S12 ^{1}H NMR spectrum of isotopic labelling experiment using $^{13}CO_2/NaH^{12}CO_3$S16

S13 Chronoamperometry of OPV photocathodes S17

S14 GC spectrum of the headspace gas in cathodic chamber S18

S15 A real-world PET bottle that was used for alkaline hydrolysis S19

S16 DSC curve of PET S20

S17 Quantitative ^{1}H NMR spectrum of PET hydrolysate S21

S18 Linear sweep voltammetry of hematite photoanodes S22

S19 Zoomed in ^{1}H NMR spectra of PET hydrolysate S23

S20 ^{1}H NMR spectrum of isophthalate S24

S21 ^{1}H NMR spectra of formate in PET hydrolysate S25

S22 Proposed reaction pathway for EG oxidation to formate S26

S23 Chronoamperometry of hematite photoanodes S26

S24 HPLC chromatograms of anolyte S27

S25 Zoomed in ^{1}H NMR spectrum of post-PEC electrolyte S28

S26 Linear sweep voltammetry of hematite photoanodes with different concentrations of PET hydrolysate S29
S27 Quantitative ^{1}H NMR spectrum of post-PEC catholyte S30

List of Tables

S1 Numerical data for CO_2 photoreduction on OPV photocathodes S31
S2 Numerical data for PET reforming on hematite photocathodes S32
S3 Comparison among state-of-the-art PEC tandem cells for solar formate production S33

S1 Photographs of Photoelectrodes

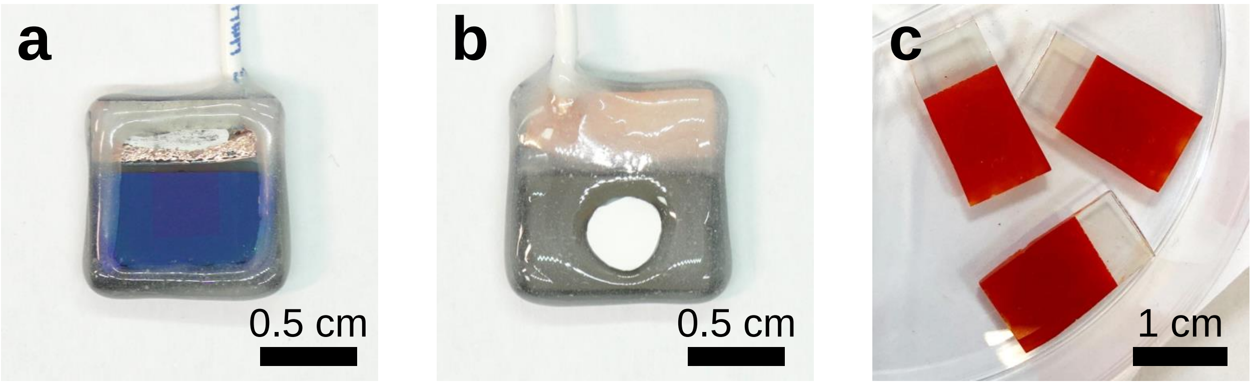

Figure S1: Photographs of an OPV photocathode from (a) the front and (b) back view. (c) Photograph of hematite photoanodes.

S2 Energy Level Diagram of All Layers in the OPV

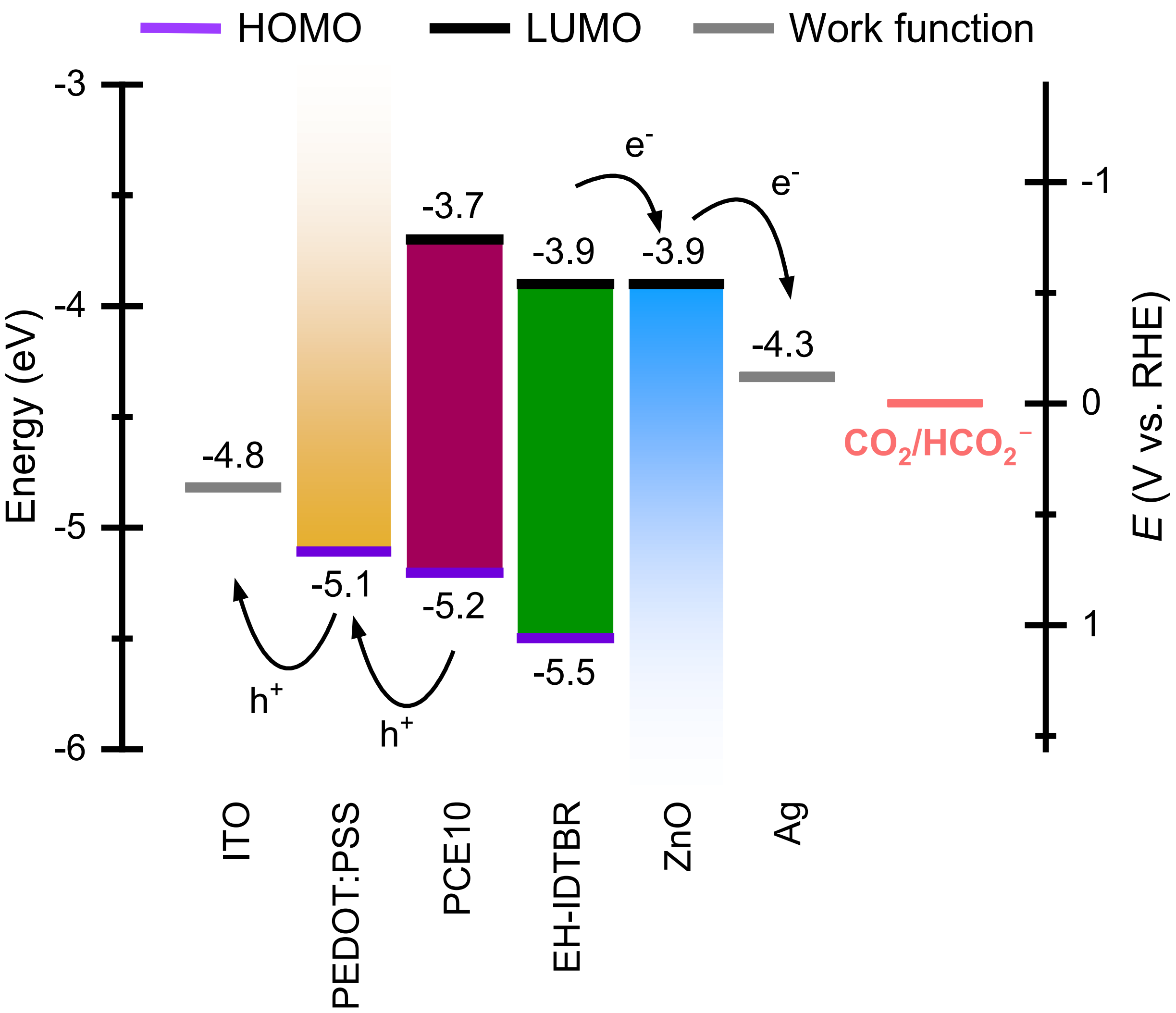

Figure S2: Energy level diagram of all layers in the OPV.

S3 OPV Performance Metrics

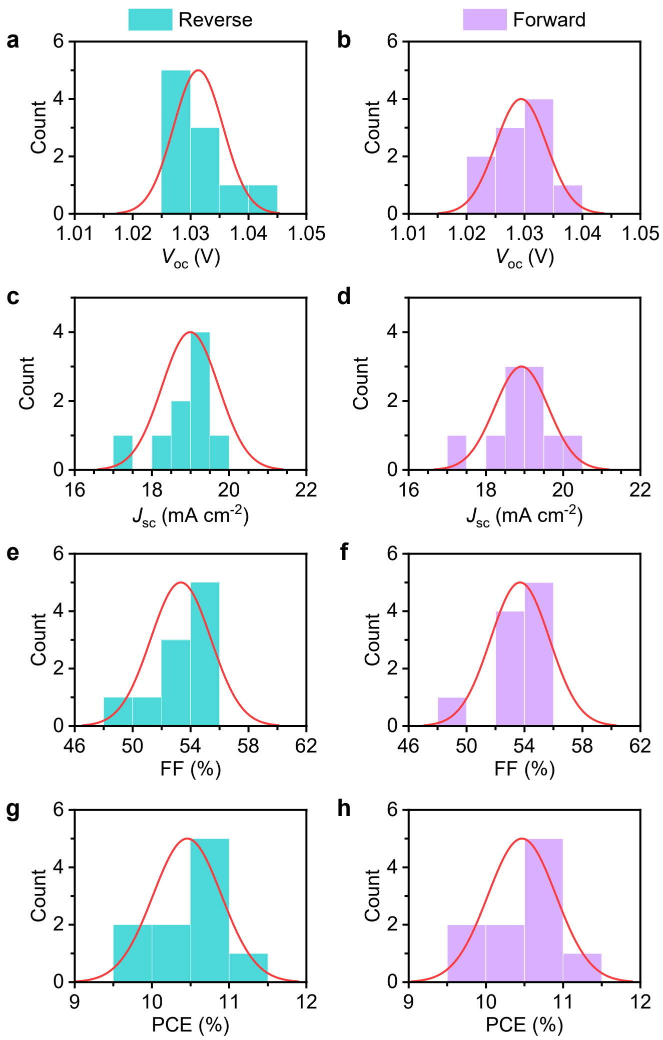

Figure S3: (a-b) Open-circuit voltage (V_{OC}). (c-d) Short-circuit current density (J_{SC}). (e-f) Fill factor (FF). (g-h) Photovoltaic cell efficiency (PCE). The blue and purple histograms denote data from the reverse and forward scans respectively, while the bold red lines represent normal distribution curves. The devices average 1.031 ± 0.004 V V_{OC}, -19.0 ± 0.7 mA cm^{-2} J_{SC}, $53.3 \pm 2.1\%$ FF and $10.5 \pm 0.4\%$ PCE in the reverse scan, and 1.029 ± 0.004 V V_{OC}, -18.9 ± 0.7 mA cm^{-2} J_{SC}, $53.7 \pm 2.0\%$ FF and $10.5 \pm 0.4\%$ PCE in the forward scan under 1 sun irradiation. Active area = 0.25 cm^2.

S4 Champion OPV Device

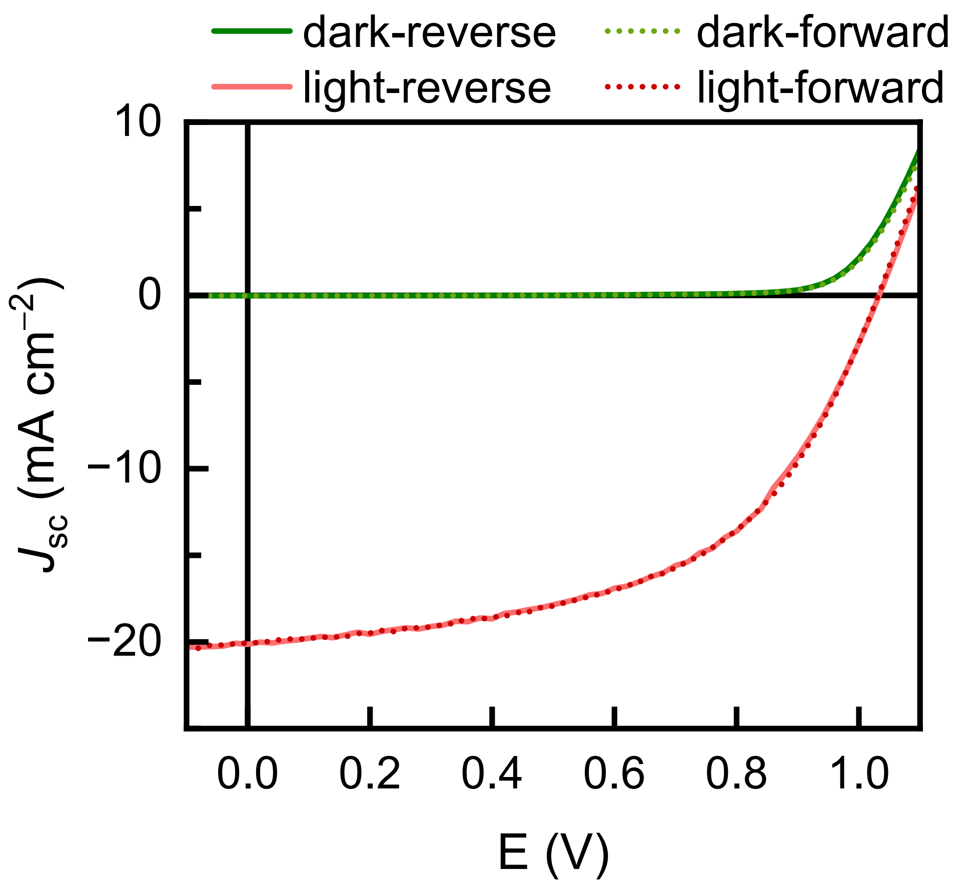

Figure S4: Forward and reverse J-V scans of the champion OPV device (active area = 0.25 cm^2). The device displayed 1.032 V V_{OC}, −20.1 mA cm^{-2} J_{SC}, 52.9% FF and 11.1% PCE in the reverse scan, and 1.031 V V_{OC}, −20.1 mA cm^{-2} J_{SC}, 53.7% FF and 11.1% PCE in the forward scan under 1 sun irradiation.

S5 Mott−Schottky Analysis of Hematite

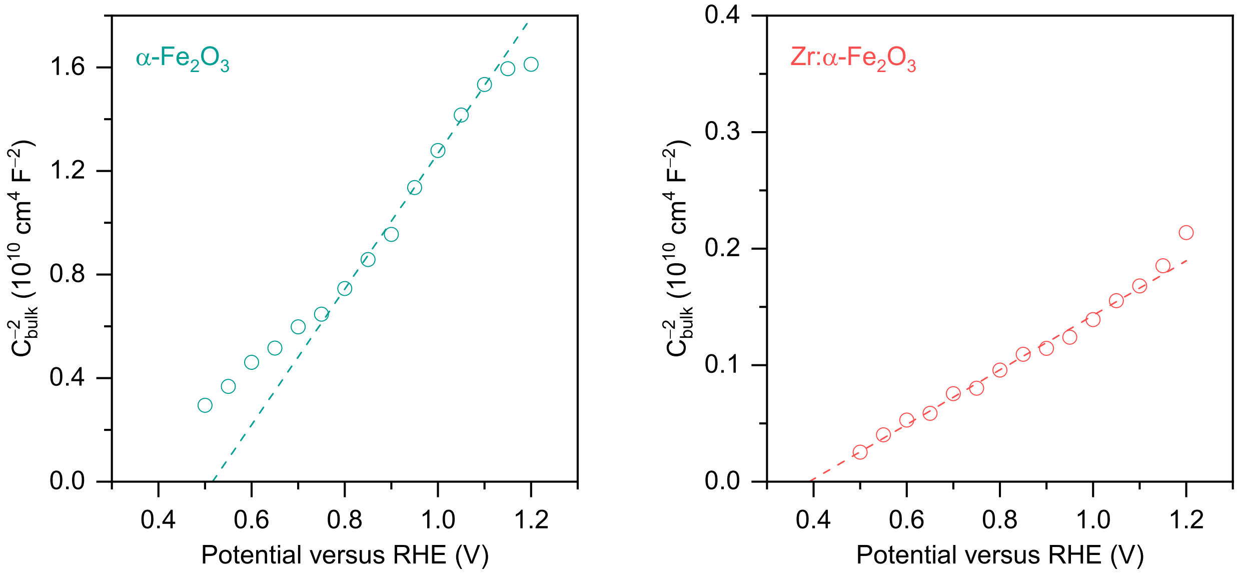

Figure S5: Mott−Schottky plots of an un-doped α-Fe_2O_3 (left) photoanode and a Zr:α-Fe_2O_3 (right) photoanode.

S6 Nyquist Plot of Hematite

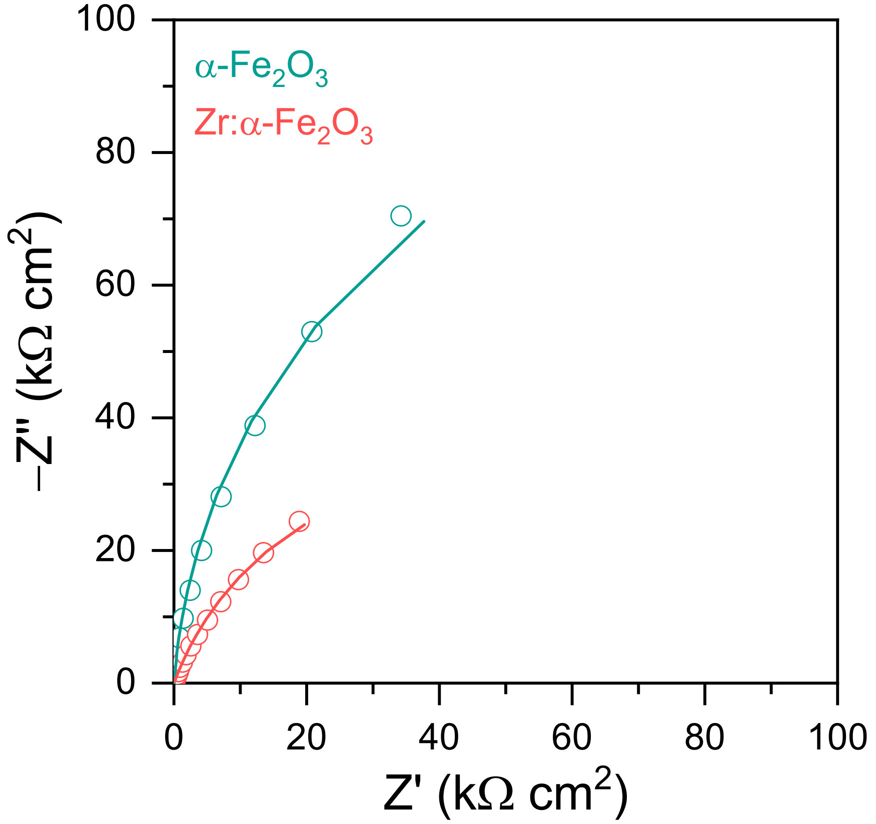

Figure S6: Nyquist plot of impedance response (open circuit) with corresponding fitting curve (solid line) using a Randles circuit. EIS was recorded at 0.5 V vs RHE in the dark.

S7 Ultraviolet–Visible (UV–Vis) Spectroscopy of Hematite

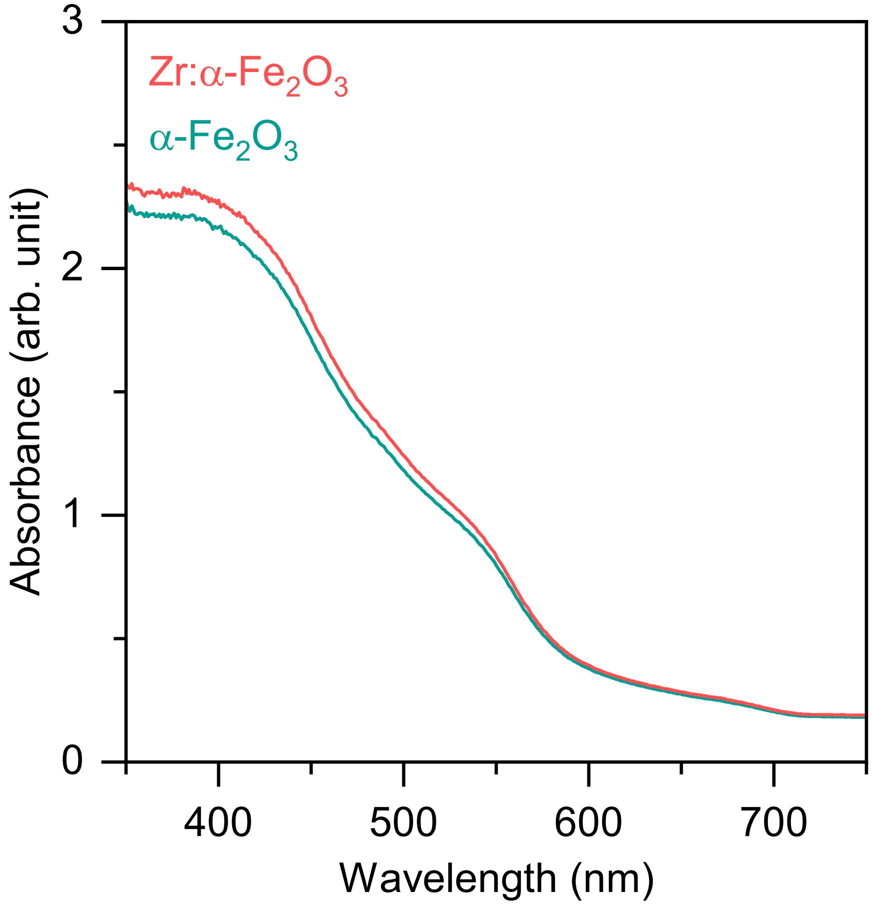

Figure S7: UV–Vis spectra of hematite photoanodes. Zr doping has minimal impact on the light absorption range, consistent with a previous report.[S1]

S8 SEM Images of Hematite

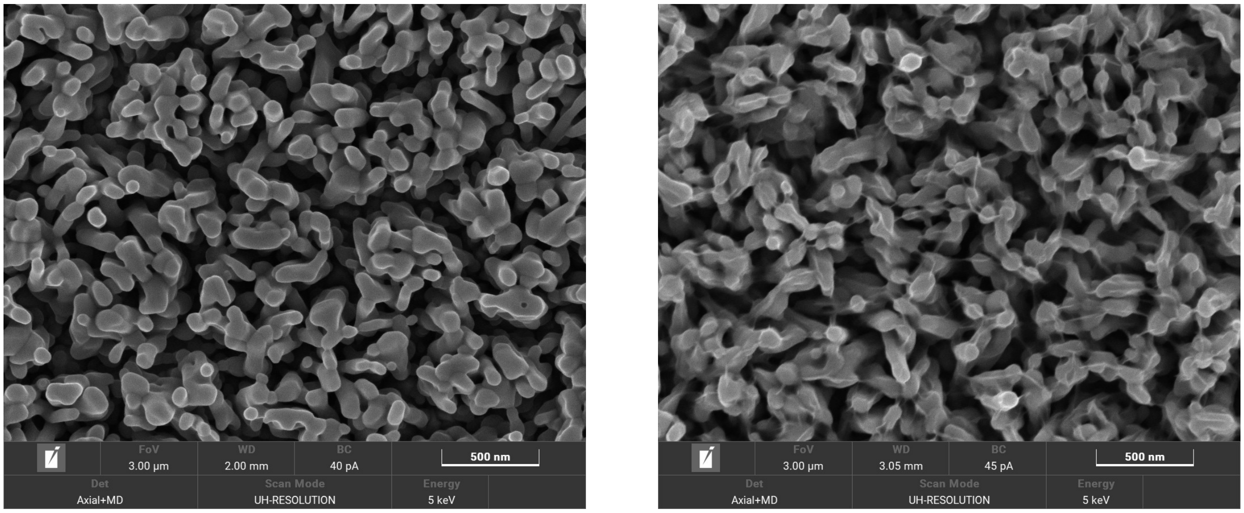

Figure S8: SEM images of Zr:α-Fe_2O_3 (left) and Zr:α-$Fe_2O_3|Ni(OH)_x$ (right).

S9 TEM Images and Elemental Mapping of Hematite

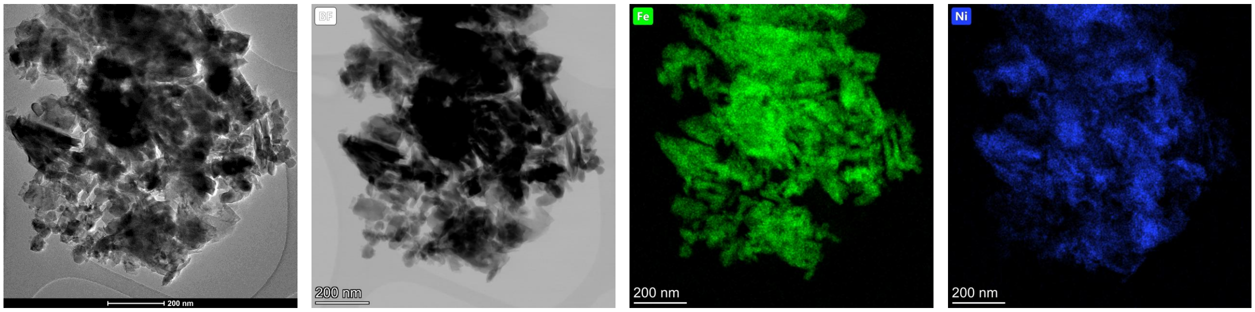

Figure S9: From left to right: TEM image, bright-field (BF) TEM image, elemental mapping of Fe (green), and Ni (blue) on hematite.

S10 XRD Patterns of Hematite

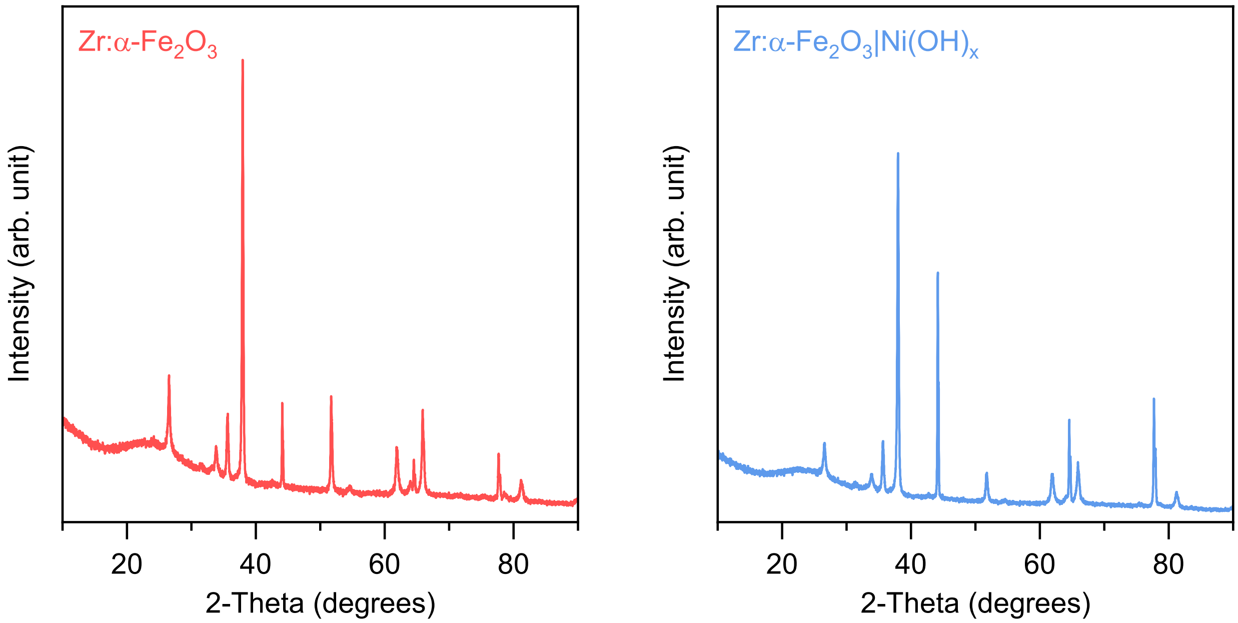

Figure S10: XRD patterns of Zr:α-Fe_2O_3 (left) and Zr:α-$Fe_2O_3|Ni(OH)_x$ (right).

S11 Linear Sweep Voltammetry of OPV Photocathodes

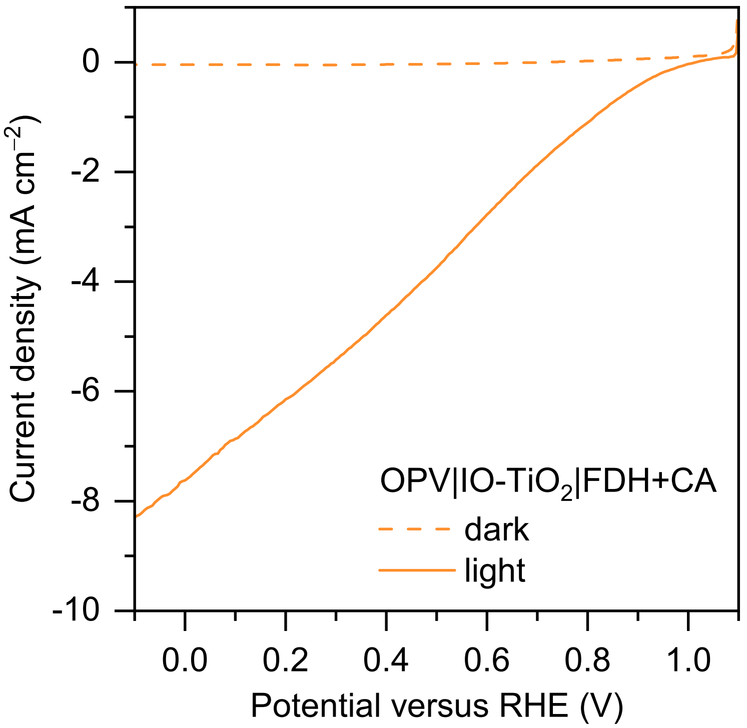

Figure S11: Linear sweep voltammetry of OPV|IO-TiO_2|FDH+CA photocathodes under constant dark and light conditions. Conditions: Simulated AM1.5G irradiation (100 mW cm^{-2}), OPV photocathode as working electrode, stirred 9 mL CO_2-saturated $NaHCO_3$ buffer (50 mM, pH 6.45) containing KCl (50 mM).

S12 Isotopic Labelling Experiment Using $^{13}CO_2/NaH^{12}CO_3$

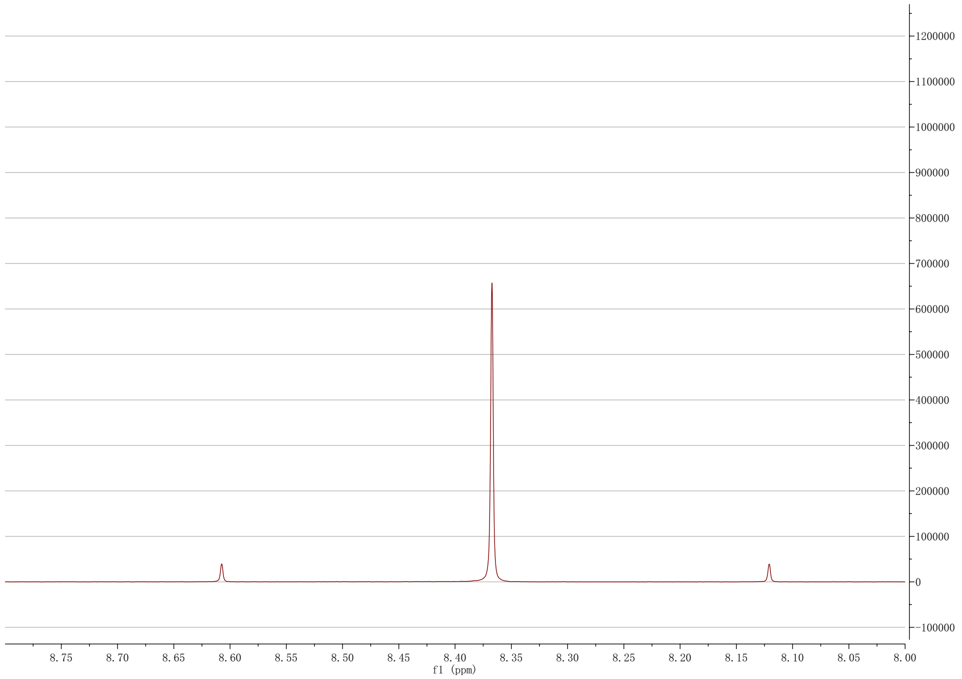

Figure S12: ^{1}H NMR spectrum of isotopic labelling experiment using $^{13}CO_2$ as the headspace in a $^{12}CO_2$-saturated $NaH^{12}CO_3$ (50 mM) solution containing KCl (50 mM), showing that ^{12}C-formate is the primary CO_2 reduction product. It has previously been demonstrated that CO_2 (rather than bicarbonate) is the substrate of FDH in electrochemical CO_2 reduction.[S2] The presence of $NaH^{12}CO_3$ in solution enables the establishment of the CO_2 hydration equilibrium: $CO_2 + H_2O \rightleftharpoons HCO_3^- + H^+$. At neutral pH, this equilibrium results in an expected CO_2(aq) to bicarbonate ratio of 1:4. In a 9 mL $NaH^{12}CO_3$ (50 mM) solution, the initial amount of $^{12}CO_2$(aq) is ∼90 µmol, which can be regenerated from ∼360 µmol $NaH^{12}CO_3$, sufficient for producing 10–20 µmol of formate. Furthermore, CO_2 consumption by FDH is continuously compensated by the rapid conversion of $H^{12}CO_3^-$(aq) to $^{12}CO_2$(aq) due to the catalytic activity of carbonic anhydrase. In contrast, at the gas–liquid interface, the dissolution and hydration of headspace $^{13}CO_2$(g) into soluble $^{13}CO_2$(aq) is slow. As a result, despite the presence of $^{13}CO_2$ in the headspace, the predominant CO_2 species in the electrolyte remains $^{12}CO_2$(aq).

S13 Chronoamperometry of OPV Photocathodes

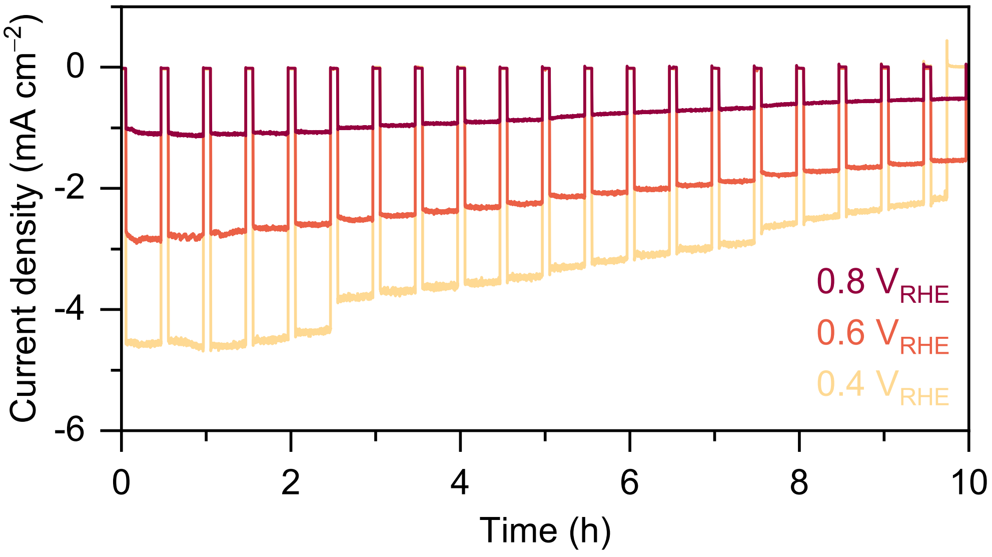

Figure S13: Chronoamperometry of OPV|IO-TiO_2|FDH+CA photocathodes. Conditions: Simulated AM1.5G irradiation (100 mW cm^{-2}), OPV photocathode as working electrode, stirred 9 mL CO_2-saturated $NaHCO_3$ buffer (50 mM, pH 6.45) containing KCl (50 mM).

S14 Gas Chromatography (GC) of the Cathodic Chamber

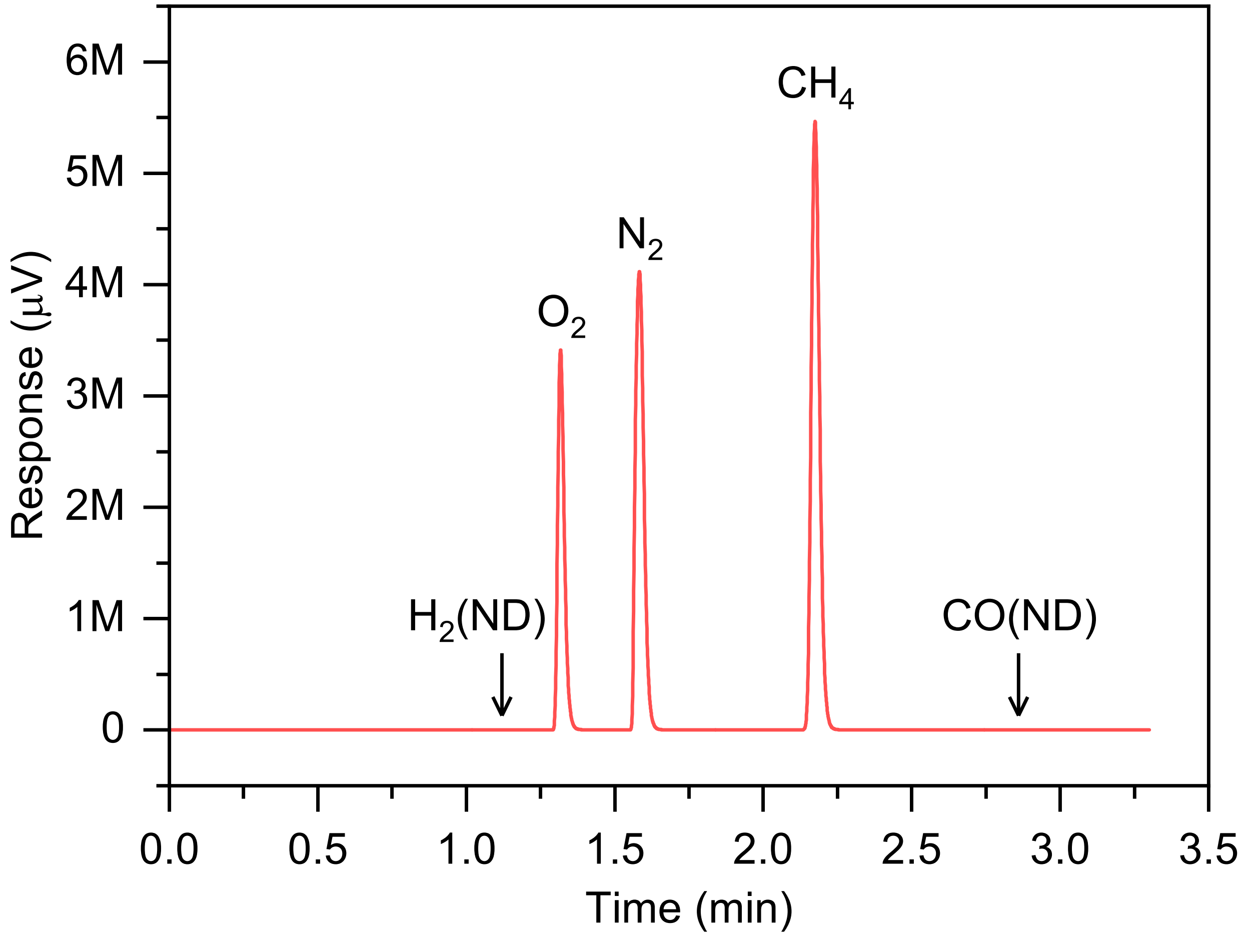

Figure S14: GC spectrum of the headspace gas in cathodic chamber after PEC CO_2 reduction. No detectable CO or H_2 was observed.

S15 A Real-World PET Bottle That Was Used for Alkaline Hydrolysis

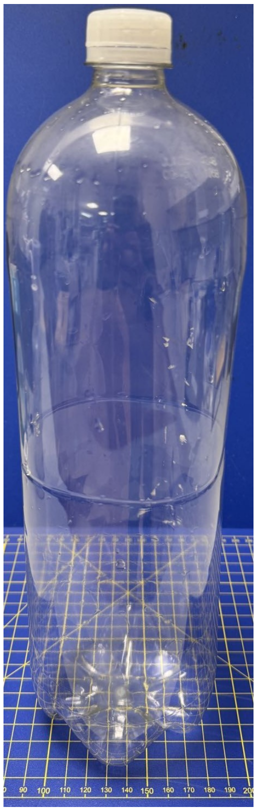

Figure S15: A real-world PET bottle that was used for alkaline hydrolysis. Grid unit: mm.

S16 Differential Scanning Calorimetry (DSC)

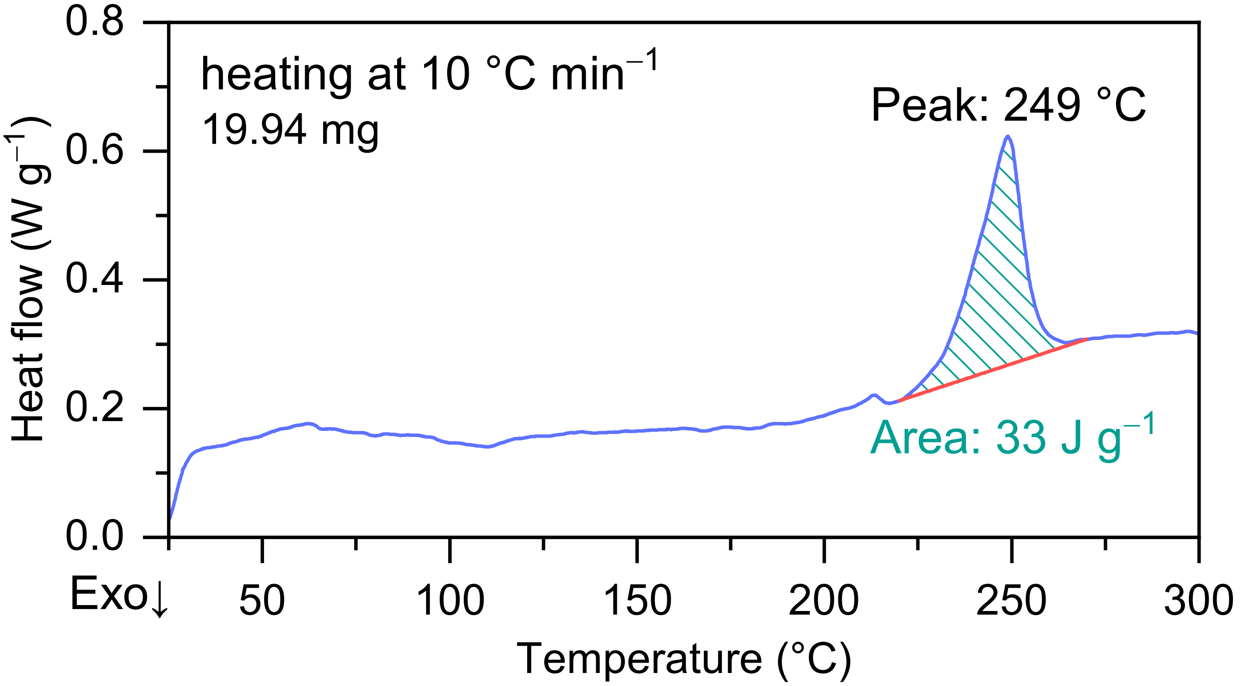

Figure S16: DSC curve of a real-world PET sample heated at a rate of 10 °C min^{-1}. Given that the heat of fusion for 100% crystalline PET is 140 J g^{-1},[S3,S4] the crystallinity is determined by calculating the ratio of the measured heat of fusion of the PET sample to 140 J g^{-1}, yielding a crystallinity of 24%.

S17 Quantitative ¹H NMR Spectrum of PET Hydrolysate

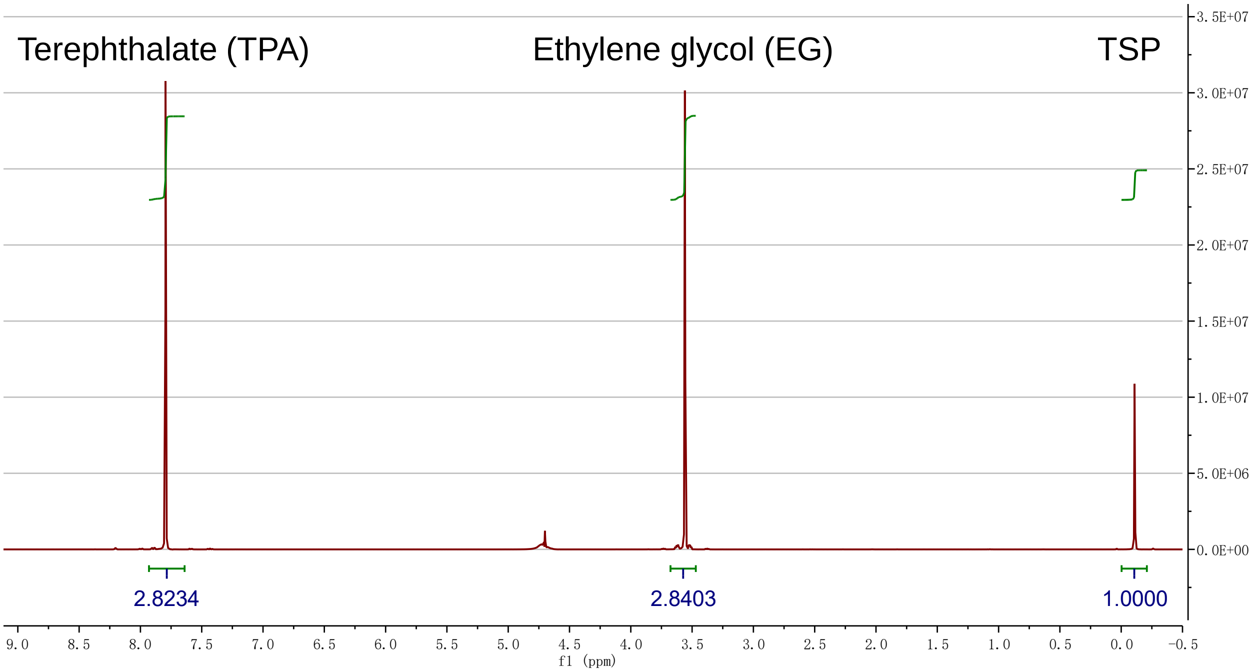

Figure S17: Quantitative ^{1}H NMR spectrum (400 MHz, D_2O) of PET hydrolysate. 3-(trimethylsilyl) propionic-2,2,3,3-d_4 acid sodium salt was added as an internal standard for quantification.

S18 Linear Sweep Voltammetry of Hematite Photoanodes

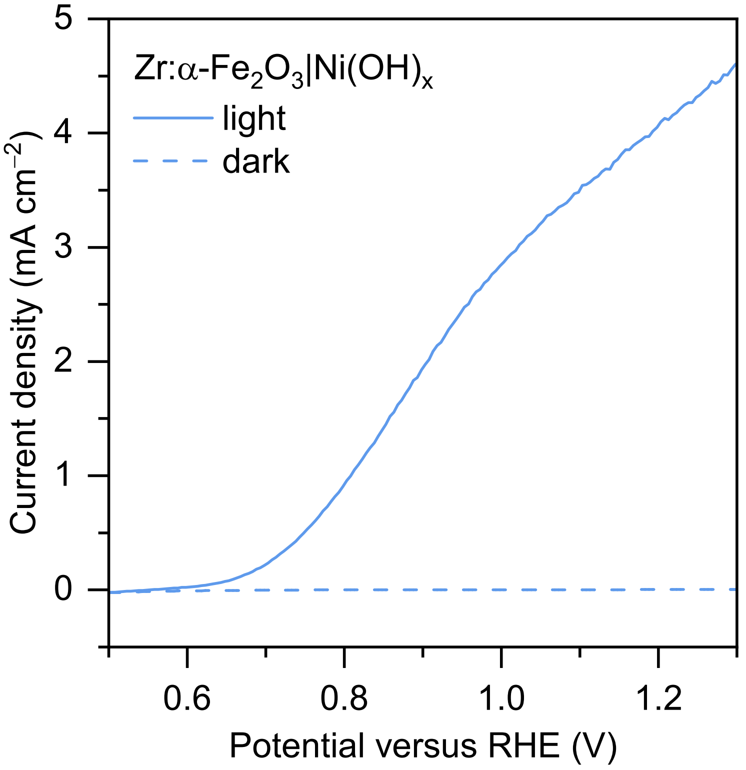

Figure S18: Linear sweep voltammetry of Zr:α-$Fe_2O_3|Ni(OH)_x$ photoanodes under constant dark and light conditions. Conditions: Simulated AM1.5G irradiation (100 mW cm^{-2}), hematite photoanode as working electrode, stirred 9 mL N_2-saturated PET hydrolysate (0.1 g mL^{-1}) in KOH (1 M, pH 14).

S19 Zoomed in ^{1}H NMR Spectra of PET Hydrolysate

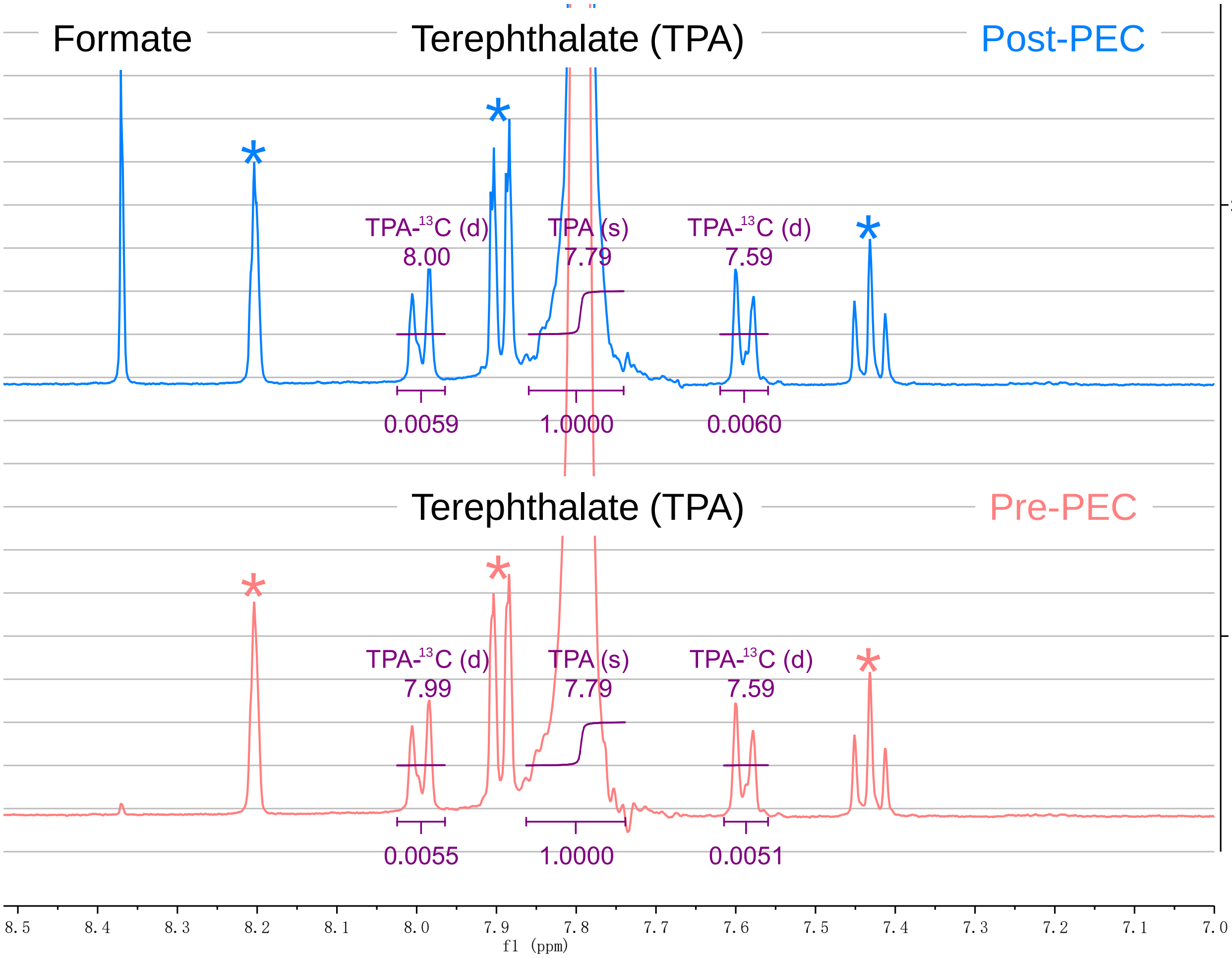

Figure S19: Zoomed in ^{1}H NMR spectra (400 MHz, D_2O) of pre- and post-PEC electrolyte on a Zr:α-Fe_2O_3|$Ni(OH)_x$ photoanode. Asterisk denotes isophthalate (Figure S20) in PET hydrolysate. The peak of TPA-^{12}C locates in the middle of the peaks of TPA-^{13}C (coupling constant = 160 Hz). The integration of TPA-^{13}C is close to the natural abundance of carbon-13 ($\sim$1.1% of carbon-12).[S5,S6] 3-(trimethylsilyl) propionic-2,2,3,3-d_4 acid sodium salt was added as an internal standard for quantification.

S20 ^{1}H NMR Spectrum of Isophthalate

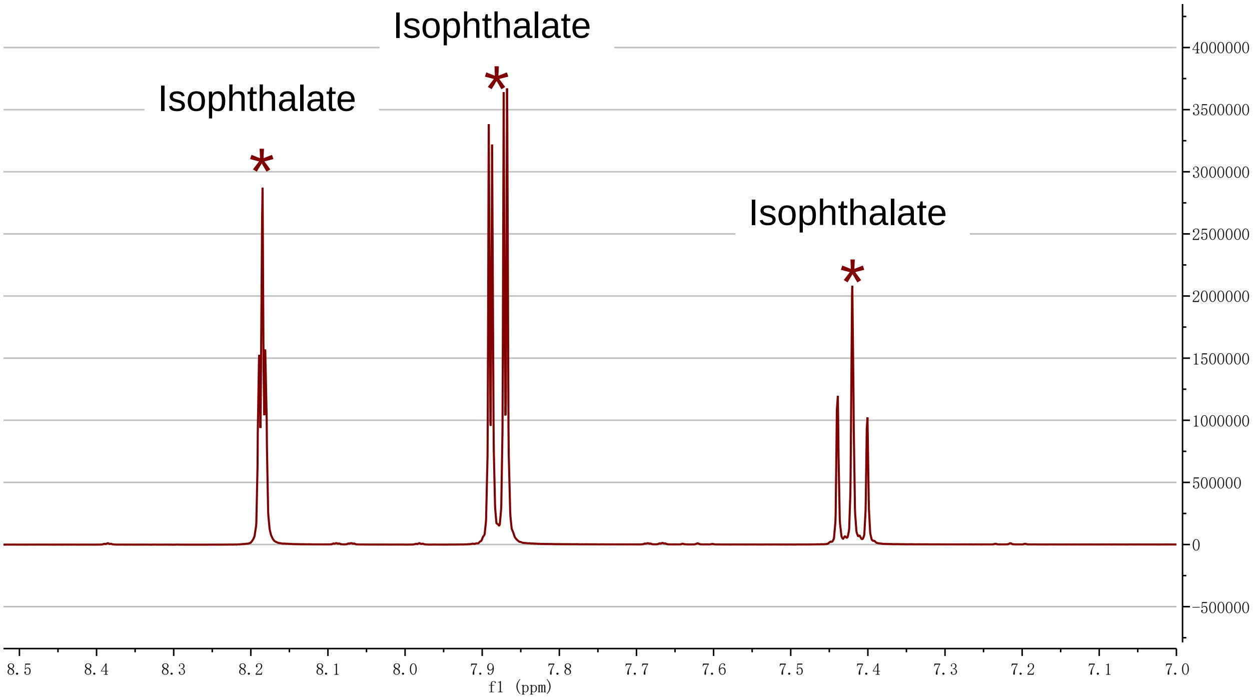

Figure S20: ^{1}H NMR spectrum (400 MHz, D_2O) of isophthalic acid dissolved in KOH (1 M, pH 14).

S21 ^{1}H NMR Spectra of Formate in PET Hydrolysate

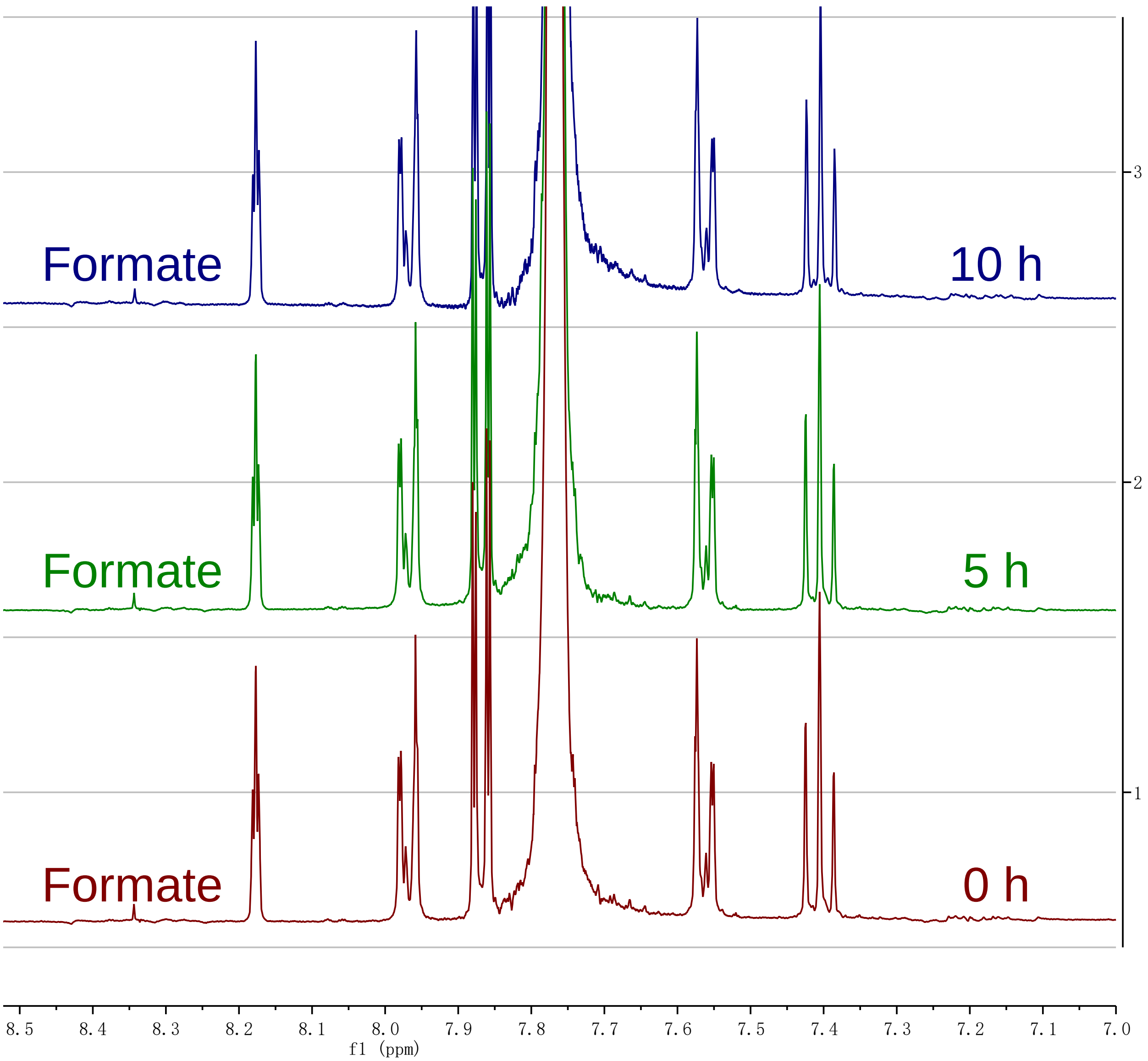

Figure S21: Superimposed ^{1}H NMR spectra (400 MHz, D_2O) of PET hydrolysate irradiated under AM1.5G in the presence of a hematite photoanode under open circuit conditions. Electrolyte aliquots were collected at 0, 5, and 10 h.

S22 Proposed Reaction Pathway for EG Oxidation to Formate

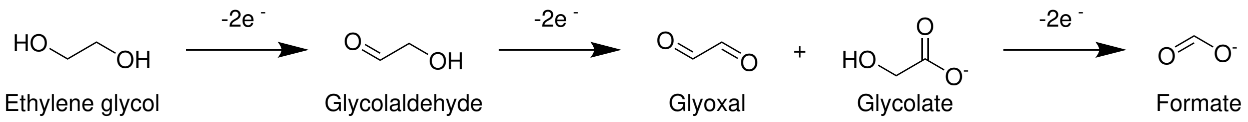

Figure S22: Proposed reaction pathway for EG oxidation to formate on a Ni-based co-catalyst in alkaline conditions.[S7]

S23 Chronoamperometry of Hematite Photoanodes

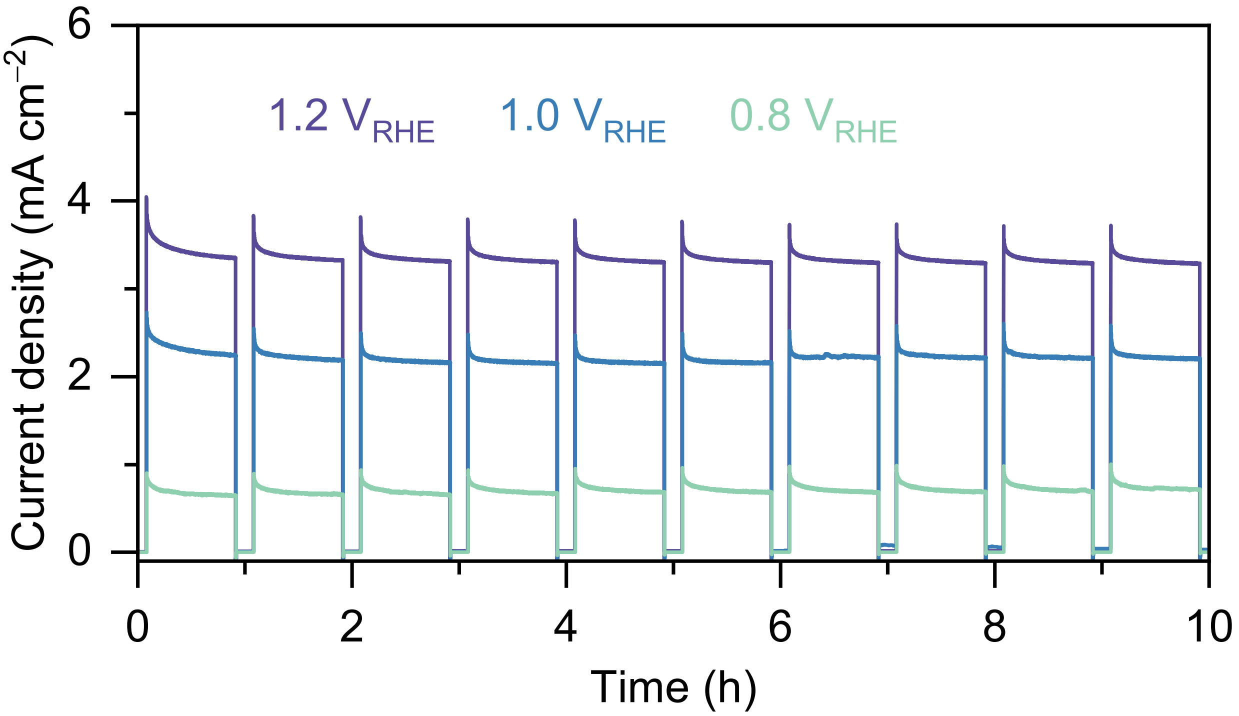

Figure S23: Chronoamperometry of Zr:α-Fe_2O_3|$Ni(OH)_x$ photoanodes. Conditions: Simulated AM1.5G irradiation (100 mW cm^{-2}), hematite photoanode as working electrode, stirred 9 mL N_2-saturated PET hydrolysate (0.1 g mL^{-1}) in KOH (1 M, pH 14).

S24 HPLC Chromatograms

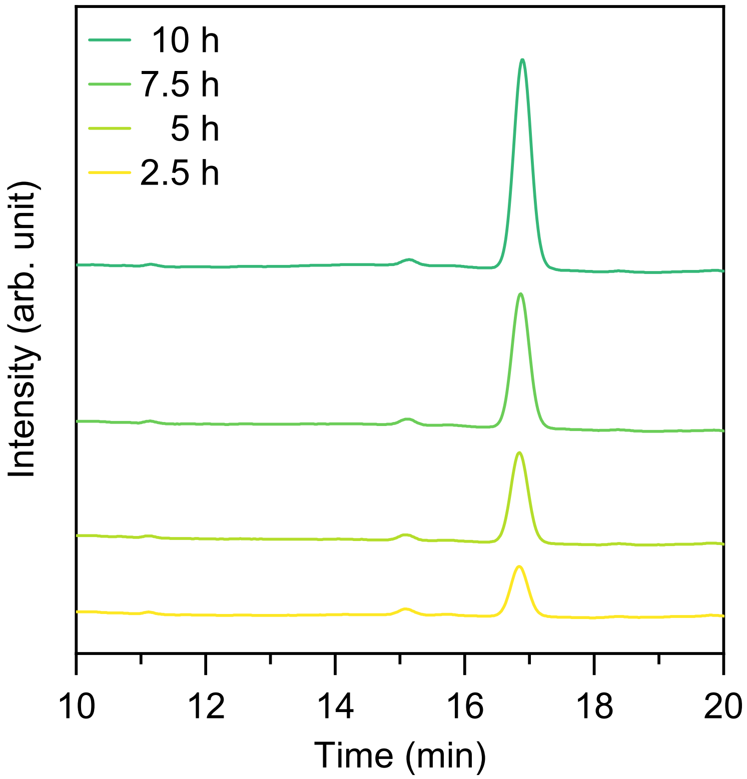

Figure S24: HPLC chromatograms of anolyte during PEC reforming of PET on a Zr:α-$Fe_2O_3|Ni(OH)_x$ photoanode at 1.0 V vs RHE. Formate has a retention time around 17 min.

S25 Zoomed in ^{1}H NMR spectrum of Post-PEC Electrolyte

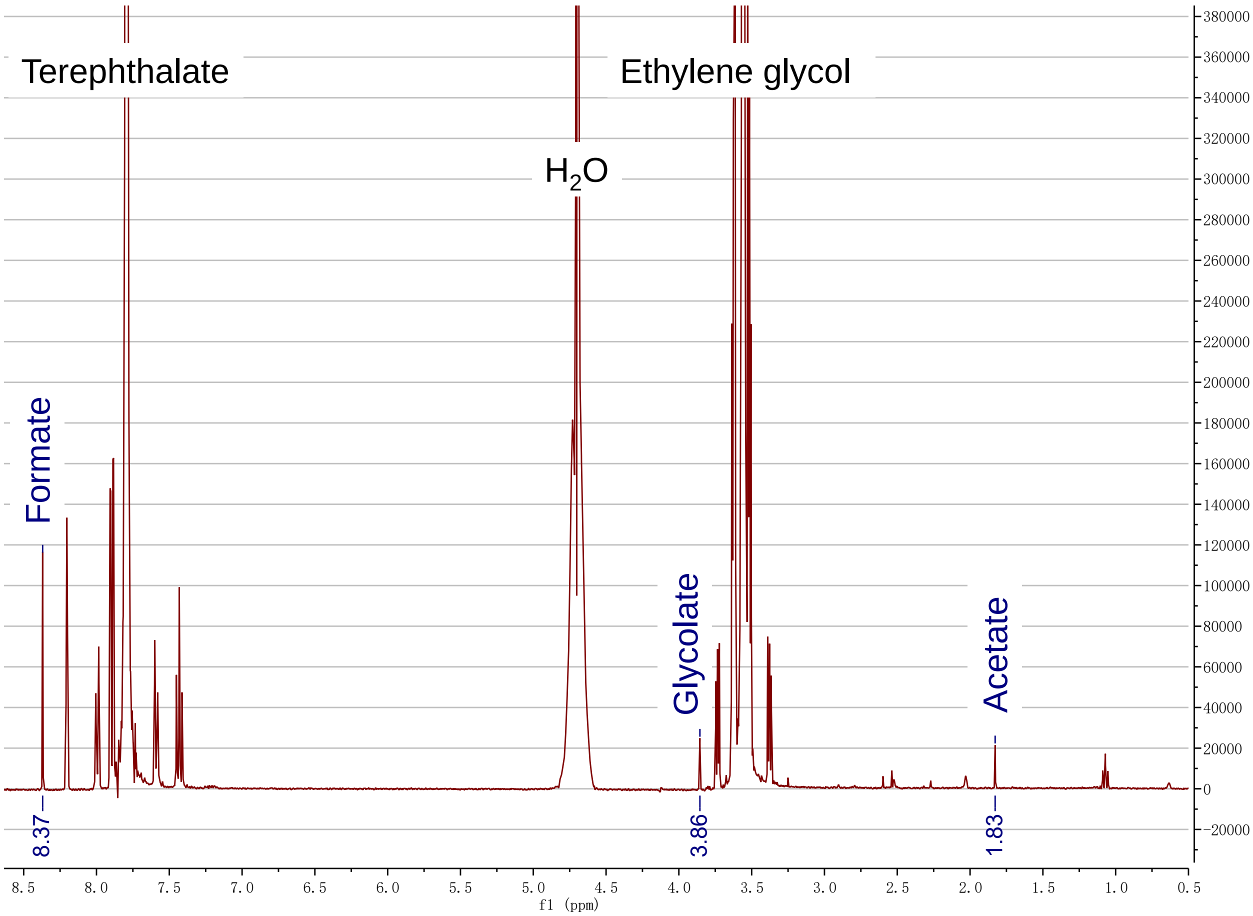

Figure S25: Zoomed in ^{1}H NMR spectrum (400 MHz, D_2O) of post-PEC electrolyte on a pristine Zr:α-Fe_2O_3 photoanode. The yield for formate, glycolate, and acetate were determined to be 6.6, 0.52, and 0.49 µmol, respectively.

S26 Linear Sweep Voltammetry of Hematite Photoanodes with Different Concentrations of PET Hydrolysate

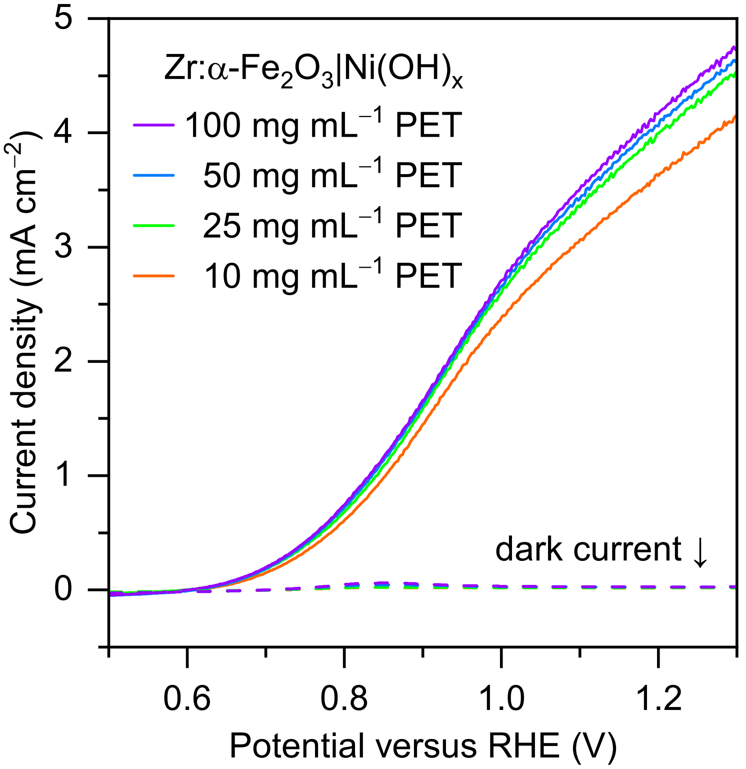

Figure S26: Linear sweep voltammetry of Zr:α-Fe_2O_3|$Ni(OH)_x$ photoanodes under constant dark and light conditions with 10, 25, 50, 100 mg mL^{-1} of PET hydrolysate. Conditions: Simulated AM1.5G irradiation (100 mW cm^{-2}), hematite photoanode as working electrode, stirred 9 mL N_2-saturated PET hydrolysate in KOH (1 M, pH 14).

S27 Quantitative ^{1}H NMR Spectrum of Post-PEC Catholyte

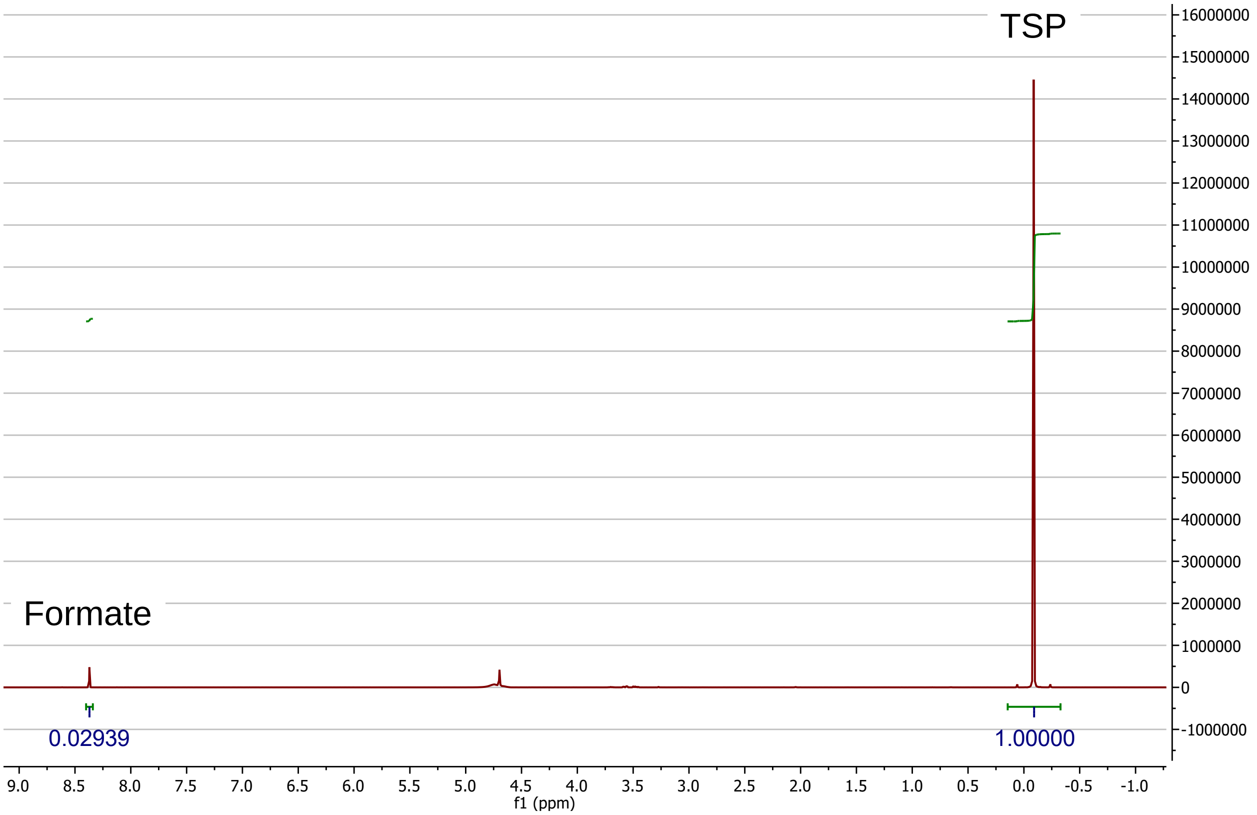

Figure S27: Quantitative ^{1}H NMR spectrum (400 MHz, D_2O) of post-PEC catholyte. 3-(trimethylsilyl) propionic-2,2,3,3-d_4 acid sodium salt was added as an internal standard for quantification.

S28 Numerical Data for CO_2 Photoreduction on OPV Photocathodes

Table S1: Numerical data for CO_2 photoreduction on OPV photocathodes. Conditions: 2-compartment electrochemical cell with a 3-elecrode configuration: an OPV—IO-TiO_2 working electrode, a Pt mesh counter electrode, a RE-6 Ag/AgCl reference electrode, and a Nafion ion exchange membrane. The CO_2-saturated electrolyte contains KCl (50 mM) and $NaHCO_3$ (50 mM, pH 6.45).

System	Potential (V_{RHE})	Time (h)	Formate yield (µmol cm^{-2})	Formate FE (%)
OPV|IO-TiO_2|FDH+CA	0.4	2.5	166.9 ± 0.9	105.7 ± 0.6
		5	282.6 ± 1.7	99.5 ± 0.6
		7.5	365.9 ± 0.2	93.3 ± 0.1
		10	413.4 ± 2.1	88.5 ± 0.5
	0.6	2.5	108.7 ± 1.3	103.1 ± 1.2
		5	196.9 ± 0.7	99.6 ± 0.4
		7.5	259.2 ± 2.3	94.1 ± 0.8
		10	304.3 ± 21.0	89.6 ± 6.2
	0.8	2.5	43.1 ± 2.0	101.8 ± 4.7
		5	73.9 ± 1.4	94.1 ± 1.8
		7.5	99.6 ± 1.4	92.7 ± 1.3
		10	114.1 ± 0.7	88.3 ± 0.5

S29 Numerical Data for PET Reforming on Hematite Photocathodes

Table S2: Numerical data for PET reforming on hematite photocathodes. Conditions: 2-compartment electrochemical cell with a 3-electrode configuration: a hematite working electrode, a Pt mesh counter electrode, a RE-61AP Hg/HgO reference electrode, and a Nafion ion exchange membrane. The N_2-saturated electrolyte is PET hydrolysate (pH 14) from the alkaline hydrolysis of real-world PET bottles.

System	Potential (V_{RHE})	Time (h)	Formate yield (μmol cm^{-2})	Formate FE (%)
Zr:α-Fe_2O_3|$Ni(OH)_x$	0.8	2.5	16.8 ± 1.2	93.6 ± 6.9
		5	34.8 ± 2.2	96.4 ± 6.1
		7.5	50.1 ± 5.2	91.5 ± 9.6
		10	64.8 ± 9.9	88.0 ± 13.4
	1.0	2.5	46.3 ± 2.3	103.7 ± 5.3
		5	85.8 ± 6.1	100.2 ± 7.1
		7.5	127.2 ± 14.7	97.9 ± 11.3
		10	163.9 ± 16.8	95.9 ± 9.8
	1.2	2.5	90.4 ± 6.1	102.4 ± 6.9
		5	176.1 ± 14.9	100.7 ± 8.5
		7.5	253.4 ± 25.2	96.9 ± 9.7
		10	330.9 ± 45.4	95.2 ± 13.1

S30 Comparison among State-of-the-art PEC Tandem Cells for Solar Formate Production

Table S3: Comparison among state-of-the-art PEC tandem cells for solar formate production.[S7–S16]

Photoelectrochemical tandem device	Reduction	Oxidation	Formate partial current density (mA cm^{-2})	Formate production rate (μmol cm^{-2} h^{-1})	Formate FE (%)	Stability (h)	Reference
FDH|NADH|mITO||CIGS|$BiVO_4$|FeOOH	CO_2R	OER	NA	0.1	NA	72	*J. Mater. Chem. A* **2020**, *8*, 8496-8502
ClFDH|$mTiO_2$|CFO|$BiVO_4$|FeOOH	CO_2R	OER	0.051	0.098	34	12	*ChemSusChem* **2020**, *13*, 2940-2944
FDH|mITO||$mTiO_2$|DPP-CA/STEMPO	CO_2R	4-MBA to 4-MBAd	0.022	0.36	74	6	*Nat. Syn.* **2022**, *1*, 77-86
ClFDH|TiN|PVK||$BiVO_4$|FeOOH	CO_2R	OER	0.074	0.78	83	8	*Adv. Energy Mater.* **2019**, *9*, 1900029
RuC|TiO_2|N,Zn:α-Fe_2O_3|Cr_2O_3||$SrTiO_{3-x}$	CO_2R	OER	0.079	1.55	79	3.1	*ACS Catal.* **2018**, *8*, 1405-1416
FDH+CA|IO-TiO_2|OPV||$BiVO_4$|TiCo	CO_2R	OER	0.539	6.3	98	10	ChemRxiv-**2024**-f49zl
RuO_x|Cu_2O||Mo:$BiVO_4$|NiCo-LDH	HER	EG to formate	0.6	4.8	40	8.3	*Angew. Chem.* **2025**, *64*, e202417648
FDH|IO-TiO_2|PVK|$BiVO_4$|TiCo	CO_2R	OER/$MOPS_{ox}$	0.664	7.1	83	10	*Angew. Chem.* **2021**, *60*, 26303-26307
FDH|IO-TiO_2|PVK||NiF|$Cu_{27}Pd_{73}$	CO_2R	PET to glycolate	0.96	12.1	96	10	*Nat. Syn.* **2023**, *2*, 182-192
Bi|GaN|Si||2×Si PV||Ti:α-Fe_2O_3|NiOOH	CO_2R	biomass to formate	1.6	23.27	160	80	*Nat. Commun.* **2023**, *14*, 1013
FDH+CA|IO-TiO_2|OPV||Zr:α-Fe_2O_3|$Ni(OH)_x$	CO_2R	PET to formate	1.5	11	176	10	**This work**

References

(S1) Kumar, P.; Sharma, P.; Shrivastav, R.; Dass, S.; Satsangi, V. R. Electrodeposited zirconium-doped α-Fe_2O_3 thin film for photoelectrochemical water splitting. *Int. J. Hydrog. Energy* **2011**, *36*, 2777–2784.

(S2) Meneghello, M.; Oliveira, A. R.; Jacq-Bailly, A.; Pereira, I. A. C.; Léger, C.; Fourmond, V. Formate dehydrogenases reduce CO_2 rather than HCO_3^-: an electrochemical demonstration. *Angew. Chem. Int. Ed.* **2021**, *60*, 9964–9967.

(S3) Wunderlich, B. *Macromolecular physics: crystal structure, morphology, defects*; Academic Press: New York and London, 1973; Vol. 1.

(S4) Wunderlich, B. *Thermal analysis of polymeric materials*; Springer, 2005.

(S5) Moutzouri, P.; Kiraly, P.; Phillips, A. R.; Coombes, S. R.; Nilsson, M.; Morris, G. A. ^{13}C satellite-free ^{1}H NMR spectra. *Anal. Chem.* **2017**, *89*, 11898–11901.

(S6) Bahadoor, A.; Brinkmann, A.; Melanson, J. E. ^{13}C-satellite decoupling strategies for improving accuracy in quantitative nuclear magnetic resonance. *Anal. Chem.* **2020**, *93*, 851–858.

(S7) Kang, F.; Wang, Q.; Du, D.; Wu, L.; Cheung, D. W. F.; Luo, J. Photoelectrochemical ethylene glycol oxidization coupled with hydrogen generation using metal oxide photoelectrodes. *Angew. Chem. Int. Ed.* **2025**, *64*, e202417648.

(S8) Kim, J.; Lee, Y. W.; Choi, E.-G.; Boonmongkolras, P.; Jeon, B. W.; Lee, H.; Kim, S. T.; Kuk, S. K.; Kim, Y. H.; Shin, B.; Park, C. B. Robust FeOOH/$BiVO_4$/Cu(In, Ga)Se_2 tandem structure for solar-powered biocatalytic CO_2 reduction. *J. Mater. Chem. A* **2020**, *8*, 8496–8502.

(S9) Kuk, S. K.; Jang, J.; Kim, J.; Lee, Y.; Kim, Y. S.; Koo, B.; Lee, Y. W.; Ko, J. W.; Shin, B.;

Lee, J.-K.; Park, C. B. CO_2-reductive, copper oxide-based photobiocathode for Z-scheme semi-artificial leaf structure. *ChemSusChem* **2020**, *13*, 2940–2944.

(S10) Antón-García, D.; Edwardes Moore, E.; Bajada, M. A.; Eisenschmidt, A.; Oliveira, A. R.; Pereira, I. A. C.; Warnan, J.; Reisner, E. Photoelectrochemical hybrid cell for unbiased CO_2 reduction coupled to alcohol oxidation. *Nat. Synt.* **2022**, *1*, 77–86.

(S11) Kuk, S. K.; Ham, Y.; Gopinath, K.; Boonmongkolras, P.; Lee, Y.; Lee, Y. W.; Kondaveeti, S.; Ahn, C.; Shin, B.; Lee, J.-K.; Jeon, S.; Park, C. B. Continuous 3D titanium nitride nanoshell structure for solar-driven unbiased biocatalytic CO_2 reduction. *Adv. Energy Mater.* **2019**, *9*, 1900029.

(S12) Sekizawa, K.; Sato, S.; Arai, T.; Morikawa, T. Solar-driven photocatalytic CO_2 reduction in water utilizing a ruthenium complex catalyst on p-type Fe_2O_3 with a multiheterojunction. *ACS Catal.* **2018**, *8*, 1405–1416.

(S13) Yeung, C. W. S.; Liu, Y.; Cobb, S. J.; Andrei, V.; Coito, A. M.; Manuel, R. R.; Pereira, I. A. C.; Reisner, E. Semi-artificial leaf interfacing organic semiconductors and enzymes for solar fuel synthesis. *ChemRxiv* **2024**, 10.26434/chemrxiv–2024–f49zl.

(S14) Edwardes Moore, E.; Andrei, V.; Oliveira, A. R.; Coito, A. M.; Pereira, I. A. C.; Reisner, E. A semi-artificial photoelectrochemical tandem leaf with a CO_2-to-formate efficiency approaching 1%. *Angew. Chem. Int. Ed.* **2021**, *60*, 26303–26307.

(S15) Bhattacharjee, S.; Rahaman, M.; Andrei, V.; Miller, M.; Rodríguez-Jiménez, S.; Lam, E.; Pornrungroj, C.; Reisner, E. Photoelectrochemical CO_2-to-fuel conversion with simultaneous plastic reforming. *Nat. Synt.* **2023**, *2*, 182–192.

(S16) Pan, Y.; Zhang, H.; Zhang, B.; Gong, F.; Feng, J.; Huang, H.; Vanka, S.; Fan, R.; Cao, Q.; Shen, M.; Li, Z.; Zou, Z.; Xiao, R.; Chu, S. Renewable formate from sunlight, biomass and carbon dioxide in a photoelectrochemical cell. *Nat. Commun.* **2023**, *14*, 1013.
